# Willingness to pay for small‐quantity lipid‐based nutrient supplements for women and children: Evidence from Ghana and Malawi

**DOI:** 10.1111/mcn.12518

**Published:** 2017-09-28

**Authors:** Katherine P. Adams, Stephen A. Vosti, Emmanuel Ayifah, Thokozani E. Phiri, Seth Adu‐Afarwuah, Kenneth Maleta, Ulla Ashorn, Mary Arimond, Kathryn G. Dewey

**Affiliations:** ^1^ Program in International and Community Nutrition, Department of Nutrition University of California, Davis Davis California USA; ^2^ Department of Agricultural and Resource Economics University of California, Davis Davis California USA; ^3^ Department of Economics, School of Economic and Business Sciences University of the Witwatersrand Johannesburg South Africa; ^4^ School of Public Health and Health Systems University of Waterloo Ontario Canada; ^5^ Department of Nutrition and Food Science University of Ghana Accra Ghana; ^6^ School of Public Health and Family Medicine University of Malawi College of Medicine Blantyre Malawi; ^7^ Department of International Health University of Tampere School of Medicine Tampere Finland

**Keywords:** demand, Ghana, Malawi, small‐quantity lipid‐based nutrient supplements, willingness to pay

## Abstract

Small‐quantity lipid‐based nutrient supplements (SQ‐LNS) are designed to enrich maternal and child diets with the objective of preventing undernutrition during the first 1,000 days. Scaling up the delivery of supplements such as SQ‐LNS hinges on understanding private demand and creatively leveraging policy‐relevant factors that might influence demand. We used longitudinal stated willingness‐to‐pay (WTP) data from contingent valuation studies that were integrated into randomized controlled nutrition trials in Ghana and Malawi to estimate private valuation of SQ‐LNS during pregnancy, postpartum, and early childhood. We found that average stated WTP for a day's supply of SQ‐LNS was more than twice as high in Ghana than Malawi, indicating that demand for SQ‐LNS (and by extension, the options for effective delivery of SQ‐LNS) may be very context specific. We also examined factors associated with WTP, including intervention group, household socioeconomic status, birth outcomes, child growth, and maternal and child morbidity. In both sites, WTP was consistently negatively associated with household food insecurity, indicating that subsidization might be needed to permit food insecure households to acquire SQ‐LNS if it is made available for purchase. In Ghana, WTP was higher among heads of household than among mothers, which may be related to control over household resources. Personal experience using SQ‐LNS was not associated with WTP in either site.

## 1 INTRODUCTION

Beyond the moral imperative to provide children with adequate nutrition to thrive throughout their life‐course, economic development hinges, in many ways, on improving maternal and early childhood nutrition. In developing country settings, morbidity and mortality rates, cognitive development, physical growth, educational obtainment, and economic productivity in adulthood have all been linked to early childhood nutritional status (Black et al., 2013; Hoddinott et al., 2013; Victora et al., 2008). Together, the short‐ and long‐term deleterious consequences of early childhood undernutrition impose considerable private and social costs (Alderman, 2010; Belli, Bustreo, & Preker, 2005; Hoddinott, Alderman, Behrman, Haddad, & Horton, 2013).

With growing consensus on undernutrition as a top global health and economic development priority (Copenhagen Consensus, 2012; Food and Agricultural Organization of the United Nations and World Health Organization, 2016), micronutrient supplementation has emerged as one component of the multipronged and multisectoral responses that will be necessary to achieve targeted reductions in maternal and early childhood undernutrition (Bhutta et al., 2013). Small‐quantity lipid‐based nutrient supplements (SQ‐LNS) are a novel vehicle to deliver micronutrients. They provide a wide range of micronutrients along with some key macronutrients, including essential fatty acids (Arimond et al., 2015). These small‐quantity (20 g/day) home fortificants, which contain vegetable oil, dried skimmed milk, peanut paste, sugar, and a vitamin–mineral mix, are designed to enrich maternal and child diets with the objective of preventing undernutrition during the first 1,000 days, from conception to age two. In some settings, recent efficacy trials showed positive effects on birth outcomes, child growth, and development (Adu‐Afarwuah et al., 2015; Adu‐Afarwuah et al., 2016; Hess et al., 2015; Iannotti et al., 2014; Mridha et al., 2016; Prado, Abbeddou, et al., 2016), but others showed no effects (Ashorn, Alho, Ashorn, Cheung, Dewey, Gondwe, et al., 2015; Ashorn, Alho, Ashorn, Cheung, Dewey, Harjunmaa, et al., 2015; Prado, Maleta, et al., 2016; Prado, Phuka, et al., 2016).

Despite their potential benefits, in developing countries many preventative innovations designed to improve health and/or nutritional outcomes, including products such as insecticide‐treated bednets and water filtration systems, suffer from low household‐level demand and high price sensitivity (Dupas, 2011). And at least in one setting, it appears that SQ‐LNS may face similar challenges: A year‐long market trial in Burkina Faso revealed low levels of demand relative to the recommended supplementation regimen of one sachet per child per day and very high price elasticity, especially for repeat purchases (Lybbert, Vosti, Adams, & Guissou, 2016).

This finding is significant because in contrast to ready‐to‐use therapeutic foods, which have historically been purchased and distributed by the international donor community for free via public channels, it is unlikely that SQ‐LNS will follow a similar path (Lybbert, 2011; Lybbert et al., 2016). This stems primarily from the intensity and recommended duration of SQ‐LNS consumption as well as the sheer size of the potential target populations of women and young children, making public procurement of SQ‐LNS at scale very resource intensive and distribution via public channels logistically complex. Instead, in settings where SQ‐LNS are likely to be effective and policymakers choose to promote them, a hybrid distribution system may emerge that reaches target consumers through both public channels and retail outlets (Lybbert, 2011). The success of hybrid distribution of SQ‐LNS will hinge on the answers to several key questions. In particular, what delivery platforms might be used to distribute SQ‐LNS to vulnerable populations? Can private consumers reasonably be expected to cover at least some of the SQ‐LNS production and distribution costs, and if so, what ratio of private consumer to socially subsidized cost would yield a price that target consumers might pay? How might policymakers and practitioners charged with reducing undernutrition design programs—targeted subsidies, social marketing campaigns, partnerships with existing health product outlets, incentives, and so forth—to help boost low private demand?

Several research activities have been undertaken to build an understanding of SQ‐LNS demand, including stated willingness to pay (WTP) in Niger (Tripp et al., 2011), stated WTP and a short‐term market simulation in Ethiopia (Segrè et al., 2015), an experimental auction in Ghana (Adams, Lybbert, Vosti, & Ayifah, 2016), and an experimental auction followed by a market trial in Burkina Faso (Lybbert et al., 2016). We assessed stated WTP for SQ‐LNS alongside randomized controlled trials in Ghana (DYAD‐G trial) and Malawi (DYAD‐M trial) undertaken by the International Lipid‐Based Nutrient Supplements (iLiNS) Project (http://ilins.org/). Several features of the data and analysis used in this study offer unique insight into WTP for SQ‐LNS. In particular, the randomized design and data collection activities of the two trials were, by design, very similar, making a cross‐site comparison of our results straightforward. Also, stark differences in the two sites in terms of socioeconomic conditions and rates of undernutrition provide contrasting yet realistic conditions under which SQ‐LNS might be distributed. This paper reports results using longitudinal stated WTP data that were collected from the same households at multiple time points during pregnancy, the first 6 months postpartum and early childhood (defined here as the period from approximately 6 to 18 months of age). During this time, some mothers and their children were provided with SQ‐LNS, whereas others were not. This allows us to assess the effect of consuming LNS on WTP and how that effect varies over time. Our objective is to characterize private valuation of SQ‐LNS over the course of recommended consumption and, using a diverse set of covariates, explore the factors associated with WTP.

**Key messages**

- In contexts where SQ‐LNS are likely to be effective and policymakers choose to promote them, SQ‐LNS could be delivered to nutritionally vulnerable women and children via a mix of public and private channels, with private consumers covering part of the production and distribution costs. Algorithms for allocating costs across stakeholder groups need to be identified and tested.
- Stated willingness to pay (WTP) for SQ‐LNS was more than twice as high in Ghana than in Malawi, suggesting that demand for and distribution strategies to deliver SQ‐LNS will be context specific.
- Personal experience using SQ‐LNS, identified via randomized assignment to treatment group, did not influence WTP.

## 2 PARTICIPANTS AND METHODS

The DYAD‐G trial took place in a busy commercial corridor stretching through the Lower Manya Krobo and Yilo Krobo districts in the Eastern Region of Ghana, approximately 70 km north of Accra, the nation's capital. Women were recruited for participation in the trial from the four main health facilities operating in the semi‐urban catchment area. On average, women who participated in the trial were approximately 27 years of age, had just over 7.5 years of formal education, and lived in food secure households (Adu‐Afarwuah et al., 2015). Recruitment for the DYAD‐M trial took place in the Mangochi district of the Southern Region of Malawi at a public hospital in the town of Mangochi, one rural hospital, and two rural health centres. Participants in the trial in Malawi were slightly younger than in Ghana (26.5 years of age on average) and had less formal schooling (average of approximately 4 years), and over a third lived in food insecure households (Ashorn, Alho, Ashorn, Cheung, Dewey, Harjunmaa, et al., 2015). In terms of the nutritional status of children under age five, in the Eastern Region of Ghana in 2014, 17% of children under age five were stunted, and 3.2% were wasted (Ghana Statistical Service, Ghana Health Service, & ICF International, 2015). In the Southern Region of Malawi, the rate of stunting among children under five was 41.8% in 2014, and 3.9% were wasted (National Statistical Office of Malawi, 2015).

At both sites, women who were less than 20 weeks of gestation at a routine visit to one of the health facilities described above were recruited for participation in the trial. Recruitment was rolling, spanning the end of 2009 to the end of 2011 in Ghana and early 2011 to mid‐2012 in Malawi. A total sample of 1,320 women in Ghana and 1,391 women in Malawi were enrolled and randomized into one of three equally sized intervention groups (the randomized trials are detailed in Adu‐Afarwuah et al., 2015 and Ashorn, Alho, Ashorn, Cheung, Dewey, Harjunmaa, et al., 2015). Women randomized to the control group received a daily iron–folic acid capsule throughout pregnancy, a component of the standard of antenatal care in Ghana and Malawi. This group also received a placebo (low‐dose calcium) capsule during the first 6 months postpartum. Women in a second group received a daily multiple micronutrient capsule throughout pregnancy and during the first 6 months postpartum. Women in the third arm received SQ‐LNS for pregnant and lactating women (SQ‐LNS‐P&L) through pregnancy and during the first 6 months postpartum, and their infants received SQ‐LNS for child consumption (SQ‐LNS‐Child) from 6 to 18 months of age. Infants of women in the capsule groups did not receive any supplementation. Table A1 of the Supporting Information shows the nutrient content of the capsules and SQ‐LNS products.

Ethical approval of the iLiNS Ghana study protocol (registered at http://clinicaltrials.gov as NCT00970866) was obtained from the ethics committees of the University of California, Davis, the Ghana Health Service, and the University of Ghana Noguchi Memorial Institute for Medical Research. Ethical approval of the iLiNS Malawi study protocol (registered at http://clinicaltrials.gov as NCT01239693) was obtained from the Research and Ethics Committee of the University of Malawi College of Medicine and by the Ethics Committee of Pirkanmaa Hospital District, Finland.

### 2.1 Data

Stated WTP data were collected from a random subsample (approximately 60% in Ghana and 45% in Malawi) of all households with women enrolled in the trial. Within a household, the WTP survey respondent was randomly assigned as either the mother participating in the trial or the head of her household, although in Malawi interviewing heads of household proved difficult, resulting in a substitution of the mother as the representative household respondent in almost all cases (approximately 94% of respondents).

WTP data were collected five times, divided into three periods for the purposes of this analysis. Shortly after the beginning of maternal supplementation, we elicited WTP for SQ‐LNS‐P&L for maternal consumption during pregnancy. At around the 35th week of gestation, we again elicited WTP for SQ‐LNS‐P&L during pregnancy. These two time points comprise the pregnancy period. Approximately 3 months after the birth of the infant, we elicited WTP for SQ‐LNS‐P&L for maternal consumption during the first 6 months postpartum, and this time point represents the postpartum period. Finally, at approximately 6 and 18 months after the birth of the infant, we elicited WTP for SQ‐LNS‐Child for child consumption. A timeline of WTP data collection is available in the Supporting Information.

Stated WTP for SQ‐LNS was elicited using a contingent valuation survey, described in detail in the Supporting Information. In short, after receiving brief information about undernutrition and nutrient supplements in general, respondents were asked to imagine SQ‐LNS were available for sale at a nearby kiosk and, bearing in mind their budget and regular expenses, were then led through a bidding tree to determine their maximum WTP. In Ghana, respondents were asked their WTP for a day's supply (one 20‐g sachet), and in Malawi, respondents were asked their WTP for a week's supply (seven 20 g sachets). For purposes of cross‐site comparison, WTP for a week's supply in Malawi was converted to a daily rate for all analyses. The starting bids, which were randomized across respondents, were set at GH¢ 0.20, GH¢ 0.50, or GH¢ 1.00 (approximately US $0.13, $0.33, or $0.66) for a day's supply in Ghana, and in Malawi, they were K100, K200, or K300 (approximately US $0.30, $0.60, or $0.90) for a week's supply. The starting bids were chosen to be comparable to the prices consumers would face when purchasing traditional or local products commonly used to improve diet quality among pregnant women and/or young children (the specific comparator product used to set starting bids in Ghana was soybean flour, commonly sold by nurses at prenatal clinics, whereas in Malawi, it was a corn–soy blend called Likuni Phala, designed for children aged 6 months and older).

Given that SQ‐LNS are meant to be consumed daily for many months, after respondents reported their maximum WTP for a day's supply, they were asked follow‐up questions to elicit WTP for the product throughout the relevant time period (i.e., throughout pregnancy or throughout the first 6 months postpartum for SQ‐LNS‐P&L and from 6 to 18 months of age for SQ‐LNS‐Child). In Ghana, we analysed both stated maximum WTP for a day's supply and stated long‐term WTP throughout the period. An error in the printing of the WTP surveys in Malawi rendered the estimates of long‐term WTP unreliable, so the Malawi analysis is limited to WTP for a day's supply.

To shed light on the factors that influence WTP for SQ‐LNS over the course of a child's critical window of nutritional vulnerability, we combined the WTP data with household demographic and socioeconomic data, maternal and child morbidity data, as well as birth outcome and child growth data. The covariates used in our regression analyses are defined below and in Table A2 in the Supporting Information, and the average value of each covariate by round is in Supporting Information Tables A4 and A5.

A household asset index was constructed using principal components analysis to combine data collected within a few months of enrolment on ownership of a set of assets, housing characteristics, and water and sanitation sources (Vyas & Kumaranayake, 2006). The Household Food Insecurity Access Scale Score is an indicator of a household's level of food insecurity and was based on the Household Food Insecurity Access Scale (Coates, Swindale, & Bilinsky, 2007). Each household was assigned a score between 0 and 27 on the basis of how frequently the household experienced each of the nine food insecurity conditions in the 4‐week period prior to the interview; a higher score indicates higher food insecurity. Maternal morbidity data were collected at biweekly home visits during pregnancy and at weekly home visits for the first 6 months postpartum. Mothers were asked to recall the number of days in the past week (Ghana) or past 2 weeks (Malawi) in which they experienced each of a range of morbidity symptoms. Child morbidity data were collected at weekly home visits from birth to 18 months of age. With the aid of a morbidity calendar, specific dates in the previous week in which the child experienced each of a range of morbidity symptoms were recorded. Among the range of maternal and child morbidity symptoms available in the data, we selected a subset to include in our analysis primarily based on two criteria: (a) a parent or guardian might correlate the morbidity symptom with the need for or side effects associated with SQ‐LNS, and (b) there was sufficient variation in the data. Morbidity variables were defined as dichotomous indicators of whether the mother or child experienced the morbidity symptom for one or more days during the reference period (reference periods defined in Table A3 of the Supporting Information).

### 2.2 Empirical methods

The random assignment of mothers and their infants to receive SQ‐LNS allowed us to assess whether gaining first‐hand experience using them had an impact on stated WTP for SQ‐LNS. For the pregnancy and child periods, where we had up to two observations per respondent, we estimated a random effects tobit model (separately for each period) for *i* = 1 , 2 , … , *N* survey respondents and for *t* = 1 , 2 rounds of WTP data collection for latent variable $y_{\mathrm{it}}^{*}$ as (Cameron & Trivedi, 2010)

(1) $$y_{\mathrm{it}}^{*}=\beta_{1}{\mathrm{LNS}}_{i}+\varphi T_{\mathrm{it}}+\alpha_{i}+\varepsilon_{\mathrm{it}}$$

where yit=yit*ifyit*>00otherwise.

The dependent variable, *y*_*it*_, was stated WTP in 2011 US dollars for respondent *i* at time *t*. It was observed at its true value if WTP was greater than zero and censored at zero otherwise. *LNS*_*i*_ was an indicator variable equal to one if the mother–infant dyad in respondent *i*'s household was randomized to receive SQ‐LNS and zero otherwise. The vector *T*_*it*_ comprised time‐varying controls (passage of time from enrolment/birth to WTP survey administration and indicators of randomized starting bid). The parameter α_*i*_ was a respondent‐level random effect, and ε_*it*_ was an idiosyncratic error. Because the error was likely correlated over time for a given respondent, standard errors were bootstrapped to account for clustering at the level of the respondent. For the postpartum period in which there was only one observation per respondent, we used a tobit model with robust standard errors to estimate the effect of intervention group on WTP with the same set of controls as in Equation (1). In all models, heterogeneity over time, by survey respondent and by maternal parity, was assessed using interactions with intervention group.

To estimate the association between stated WTP and the predicted correlates for the pregnancy and child periods, we extended Equation (1) to include a set of time‐varying covariates (e.g., maternal and child morbidity) in the vector *X*_*it*_, and time‐invariant covariates (e.g., infant gender) in the vector *Z*_*i*_ and estimated the following random effects tobit model

(2) $$y_{\mathrm{it}}^{*}=\beta_{1}{\mathrm{LNS}}_{i}+\theta X_{\mathrm{it}}+\delta Z_{i}+\varphi T_{\mathrm{it}}+\alpha_{i}+\varepsilon_{\mathrm{it}}$$

The vector *T*_*it*_ again comprised time‐varying controls, in this case the passage of time from enrolment to WTP survey administration (pregnancy period) or birth to WTP survey administration (postpartum and child periods), the total number of days in the morbidity reference periods, and indicators of randomized starting bid. For the postpartum period, we estimated the correlates of WTP using a tobit model with robust standard errors.

We also assessed the sensitivity of our regression results to the way the morbidity variables were defined (dichotomous vs. continuous) and the length of the morbidity reference periods. Sensitivity analysis results for each period are available in the Supporting Information.

## 3 RESULTS

### 3.1 Average WTP

Table 1 summarizes average WTP by site and by period and round. In Ghana, average WTP for a day's supply of SQ‐LNS during the pregnancy period was $0.57 and $0.51 in the first and second rounds, respectively, $0.45 in the postpartum period, and $0.39 in both the first and second rounds of the child period. Average long‐term WTP for a day's supply of SQ‐LNS throughout pregnancy was $0.46 in the first round and $0.38 in the second round. Long‐term WTP throughout the postpartum period was $0.36 on average, and average long‐term WTP throughout the time when the child was 6–18 months was $0.31 at both rounds during the child period. At each round, less than 7% of respondents indicated that they would not be willing to pay anything for SQ‐LNS. It should be noted, though, that average WTP for a day's supply of SQ‐LNS‐P&L was significantly lower in the second pregnancy period than the first and lower still in the postpartum period. Long‐term WTP throughout the pregnancy period was significantly lower at the second round than at the first, although there was no significant difference in average WTP between the second pregnancy round and the postpartum period. For both WTP for a day's supply and long‐term WTP, average WTP for SQ‐LNS‐Child was lower than WTP for SQ‐LNS‐P&L in the postpartum (and pregnancy) period.

**Table 1:** Average WTP for SQ‐LNS (US dollars)

		Ghana				Malawi			
	Period and round	*N*	Mean^a^	*SD*	% Zero WTP	*N*	Mean^a^	*SD*	% Zero WTP
Day's supply	Pregnancy 1	586	0.57	0.53	6	570	0.16	0.16	23
	Pregnancy 2	432	0.51**	0.56	6	391	0.22**	0.52	19
	Postpartum 1	538	0.45**	0.38	5	376	0.18	0.18	17
	Child 1	308	0.39***	0.35	5	448	0.16	0.23	16
	Child 2	442	0.39	0.34	2	299	0.11***	0.12	22
Long‐term throughout period	Pregnancy 1	585	0.46	0.46	6				
	Pregnancy 2	428	0.38***	0.35	7				
	Postpartum 1	538	0.36	0.31	5				
	Child 1	308	0.31***	0.26	6				
	Child 2	441	0.31	0.22	2				

*Note*. At each round, observations >6 *SD* above the mean were omitted as outliers. Differences in average WTP between rounds were assessed by testing for a significant coefficient on an indicator variable for round using ordinary least squares (OLS) with cluster‐robust standard errors. Regressions were run separately for each pair of rounds. *SD* = standard deviation; WTP = willingness to pay.

a In fourth quarter 2011 US dollars.

Significance codes indicating difference in mean from previous round:

*** (*p* < .01);

** (*p* < .05);

* (*p* < .1).

Average stated WTP for a day's supply of SQ‐LNS in Malawi was substantially lower (between 52% and 77% lower across all periods and rounds) than in Ghana. During the pregnancy period, average WTP was $0.16 in the first round and $0.22 in the second round. Average WTP for a day's supply was $0.18 in the postpartum period, and it was $0.16 and $0.11 in the first and second rounds of the child period, respectively. In contrast to Ghana, average WTP for a day's supply of SQ‐LNS‐P&L in Malawi was significantly higher in the second pregnancy round compared to the first round, although average WTP for a day's supply of SQ‐LNS‐Child was lower in the second round compared to the first in Malawi. Across all periods and rounds, between 17% and 23% of respondents indicated that they would not be willing to pay anything for SQ‐LNS.

### 3.2 Effect of experience using SQ‐LNS on WTP

There were no statistically significant differences in average WTP between the LNS group and the capsules group at any time point in either Ghana or Malawi (Supporting Information Figure A3). Our tobit model estimates confirmed no difference in WTP by intervention group in any period or over time in either Ghana or Malawi (see Supporting Information Sections 5 and 6).

### 3.3 Factors associated with WTP during the pregnancy period

Table 2 presents estimates of the association between a wide range of variables that described households' circumstances during the pregnancy period and stated willingness to pay for SQ‐LNS during that period.

**Table 2:** Factors associated with WTP during the pregnancy period

	Ghana		Malawi
	Day's supply	Long term	Day's supply
LNS group (0/1)	−0.006	0.024	0.010
	(0.045)	(0.034)	(0.039)
Months from enrolment	−0.016	−0.025***	0.027**
	(0.011)	(0.008)	(0.014)
Lean season	−0.008	−0.017	0.102***
	(0.031)	(0.021)	(0.038)
Mangochi (0/1)			0.006
			(0.027)
Mother (0/1)	−0.145**	−0.081*	
	(0.056)	(0.042)	
Respondent age	−0.005**	−0.002	0.004
	(0.002)	(0.002)	(0.004)
Respondent education	0.003	0.006	−0.003
	(0.006)	(0.005)	(0.007)
Asset index	−0.032	−0.010	0.021
	(0.027)	(0.022)	(0.018)
HFIAS score	−0.015***	−0.015***	−0.009**
	(0.005)	(0.003)	(0.004)
Primiparity (0/1)	−0.006	−0.025	−0.043
	(0.049)	(0.039)	(0.036)
Maternal poor appetite (0/1)	0.019	0.027	0.012
	(0.054)	(0.038)	(0.043)
Maternal nausea/vomiting (0/1)	−0.079*	−0.091***	−0.019
	(0.041)	(0.031)	(0.039)
Maternal diarrhoea (0/1)	0.038	0.017	−0.026
	(0.040)	(0.026)	(0.037)
Constant	0.833***	0.599***	−0.004
	(0.140)	(0.115)	(0.086)
*N*	971	966	889
Wald chi^2^	38.212	98.550	27.510

*Note*. Dependent variables are stated WTP in 2011 US dollars. The variable “Mother” = 1 if the respondent to the WTP survey was the mother participating in the randomized trial and zero if the respondent was the head of household. Controls for randomized starting bid are included in all models (unreported). Standard errors, in parentheses, obtained via 50 bootstrap replications. HFIAS = Household Food Insecurity Access Scale; LNS = lipid‐based nutrient supplements; WTP = willingness to pay.

Significance codes:

*** (*p* < .01);

** (*p* < .05);

* (*p* < .1).

In Ghana, we found that with each additional month since trial enrolment, long‐term WTP throughout the pregnancy period was approximately $0.03 lower (*p* = .001). On average, WTP among mothers was lower than among heads of household both for a day's supply ($0.15 lower, *p* = .01) and in the long term ($0.08 lower, *p* = .05). A household's food security status was also a significant predictor of WTP in Ghana, with respondents from more food insecure households indicating a lower WTP. Finally, if a mother experienced nausea or vomiting during the reference period, WTP for a day's supply was $0.08 lower (*p* = .05) and long‐term WTP was $0.09 lower (*p* = .004) than if the mother did not experience nausea or vomiting. The coefficients on the other two indicators of maternal morbidity—poor appetite and diarrhoea—were positive although not significant.

In Malawi, each additional month since enrolment was associated with a $0.03 higher (*p* = .05) WTP for a day's supply of SQ‐LNS during the pregnancy period. If the WTP survey was conducted during the lean season, a time when food is typically relatively scarce and prices higher, WTP was approximately $0.10 higher (*p* = .01) than if the WTP survey was conducted outside of the lean season. However, household food insecurity was negatively associated (*p* = .03) with WTP during the pregnancy period, with a lower WTP among more food insecure households, although the magnitude of the association was small.

### 3.4 Factors associated with WTP during the postpartum period

Shown in Table 3, WTP for SQ‐LNS during the postpartum period in Ghana was lower among mothers than heads of household and among more food insecure households. Poor infant appetite was marginally negatively associated with WTP for a day's supply (*p* = .06), and infant vomiting was marginally positively associated with WTP for a day's supply (*p* = .09). If the mother's infant was male, WTP for a day's supply of SQ‐LNS (for maternal consumption) was approximately $0.10 higher (*p* = .01) than if the infant was female, and long‐term WTP was $0.05 higher (*p* = .09) if the infant was male. Infant fatness at birth, measured as body mass index‐for‐age *z*‐score (BMIZ), was also positively and significantly associated with WTP for both a day's supply of SQ‐LNS (*p* = .01) and over the long term during the postpartum period (*p* = .01).

**Table 3:** Factors associated with WTP during the postpartum period

	Ghana		Malawi
	Day's supply	Long term	Day's supply
LNS group (0/1)	0.024	0.014	0.032
	(0.038)	(0.030)	(0.025)
Months from birth	0.036	0.012	−0.045*
	(0.023)	(0.021)	(0.025)
Lean season	0.041	0.039	−0.022
	(0.035)	(0.029)	(0.023)
Mangochi (0/1)			0.008
			(0.026)
Mother (0/1)	−0.143***	−0.091***	
	(0.041)	(0.035)	
Respondent age	−0.002	−0.001	0.003
	(0.002)	(0.001)	(0.002)
Respondent education	0.000	0.002	0.001
	(0.005)	(0.004)	(0.004)
Asset index	−0.001	0.002	0.027**
	(0.018)	(0.015)	(0.014)
HFIAS score	−0.017***	−0.019***	−0.003
	(0.006)	(0.004)	(0.003)
Primiparity (0/1)	−0.026	−0.015	0.021
	(0.035)	(0.029)	(0.039)
Maternal poor appetite (0/1)	−0.038	−0.034	−0.024
	(0.048)	(0.042)	(0.035)
Maternal diarrhoea (0/1)	0.042	0.077	−0.026
	(0.065)	(0.059)	(0.034)
Infant ill (0/1)	0.040	0.040	−0.016
	(0.046)	(0.035)	(0.024)
Infant poor appetite (0/1)	−0.090*	−0.062	0.051
	(0.047)	(0.040)	(0.031)
Infant diarrhoea (0/1)	−0.020	−0.007	0.001
	(0.041)	(0.033)	(0.027)
Infant vomiting (0/1)	0.079*	−0.018	0.005
	(0.048)	(0.039)	(0.026)
Infant male (0/1)	0.094***	0.047*	0.027
	(0.034)	(0.028)	(0.023)
BMIZ at birth	0.044**	0.036***	−0.006
	(0.017)	(0.013)	(0.011)
LAZ at birth	−0.014	−0.020	−0.005
	(0.019)	(0.015)	(0.012)
Constant	0.461***	0.392***	0.217**
	(0.141)	(0.121)	(0.102)
*N*	526	526	338
Pseudo *R* ^2^	0.095	0.132	1.373

*Note*. Dependent variables are stated WTP in 2011 US dollars. The variable “Mother” = 1 if the respondent to the WTP survey was the mother participating in the randomized trial and zero if the respondent was the head of household. Controls for randomized starting bid are included in all models (unreported). Robust standard errors in parentheses. BMIZ = Body mass index‐for‐age z‐score; HFIAS = Household Food Insecurity Access Scale; LAZ = length‐for‐age z‐score; LNS = lipid‐based nutrient supplements; WTP = willingness to pay.

Significance codes:

*** (*p* < .01);

** (*p* < .05);

* (*p* < .1).

In Malawi, for each additional month from the birth of the infant to WTP survey administration, WTP for a day's supply of SQ‐LNS was approximately $0.05 lower (*p* = .08), and the association between the household asset index and WTP was positive and significant (*p* = .05).

### 3.5 Factors associated with WTP during the child period

In the child period in Ghana, WTP for SQ‐LNS for the infant, both for a day's supply and in the long term, was lower among mothers compared to heads of household (Table 4). The association with household food insecurity was negative and significant. Finally, WTP was, on average, approximately $0.09 higher (*p* = .004) for male children than for female children, and WTP in the long term was approximately $0.05 higher (*p* = .01) for male children than for female children. None of the child morbidity variables were systematically associated with WTP in Ghana nor were the measures of child nutritional status (length‐for‐age z‐score (LAZ) and weight‐for‐length z‐score (WLZ)).

**Table 4:** Factors associated with WTP during the child period

	Ghana		Malawi
	Day's supply	Long term	Day's supply
LNS group (0/1)	0.002	−0.007	−0.015
	(0.034)	(0.019)	(0.020)
Months from birth	−0.005	−0.005	−0.010***
	(0.006)	(0.004)	(0.002)
Lean season	0.020	−0.001	−0.034
	(0.030)	(0.015)	(0.021)
Mangochi (0/1)			0.025
			(0.026)
Mother (0/1)	−0.122***	−0.086***	
	(0.030)	(0.025)	
Respondent age	0.000	−0.000	0.002
	(0.002)	(0.001)	(0.002)
Respondent education	0.004	0.003	−0.004
	(0.003)	(0.003)	(0.004)
Asset index	−0.009	0.003	0.040**
	(0.013)	(0.009)	(0.016)
HFIAS score	−0.015***	−0.011***	−0.006***
	(0.004)	(0.002)	(0.002)
Primiparity (0/1)	−0.029	−0.029	0.016
	(0.035)	(0.020)	(0.021)
Infant ill (0/1)	−0.046	−0.012	0.059**
	(0.060)	(0.032)	(0.023)
Infant poor appetite (0/1)	0.006	−0.001	0.016
	(0.042)	(0.030)	(0.018)
Infant diarrhoea (0/1)	−0.020	−0.008	−0.052**
	(0.031)	(0.019)	(0.025)
Infant vomiting (0/1)	0.025	0.010	−0.013
	(0.031)	(0.019)	(0.025)
Infant male (0/1)	0.091***	0.049***	−0.009
	(0.032)	(0.018)	(0.020)
LAZ	−0.006	0.007	−0.003
	(0.016)	(0.013)	(0.007)
WLZ	0.002	0.008	0.013
	(0.012)	(0.007)	(0.013)
Constant	0.433***	0.408***	0.182***
	(0.102)	(0.091)	(0.066)
*N*	732	731	710
Wald chi^2^	93.387	165.807	95.381

*Note*. Dependent variables are stated WTP in 2011 US dollars. The variable ‘Mother’ = 1 if the respondent to the WTP survey was the mother participating in the randomized trial and = 0 if the respondent was the head of household. Controls for randomized starting bid are included in all models (unreported). Standard errors, in parentheses, obtained via 50 bootstrap replications. HFIAS = Household Food Insecurity Access Scale; LAZ = length‐for‐age z‐score; LNS = lipid‐based nutrient supplements; WLZ = weight‐for‐length z‐score; WTP = willingness to pay.

Significance codes:

*** (*p* < .01);

** (*p* < .05);

* (*p* < .1).

In Malawi, the association between months from the birth of the infant and WTP was negative and significant (*p* < .001). There was a positive and significant (*p* = .01) association between WTP for a day's supply of SQ‐LNS for the child and the asset index and a negative association (*p* = .01) between household food insecurity and WTP. If the infant was reported to be generally ill at least once during the reference period before WTP data collection, WTP for a day's supply of SQ‐LNS was, on average, approximately $0.06 higher (*p* = .01), while infant with diarrhoea was associated with a $0.05 lower (*p* = .04) WTP.

## 4 DISCUSSION

Household choices, such as the choice to invest in a nutrient supplement such as SQ‐LNS, may be shaped by household characteristics, preferences, and constraints as well as the perceived and expected costs and benefits associated with SQ‐LNS and competing investments. The empirical results presented here, based on a pair of contingent valuation studies integrated into randomized controlled nutrition trials in Ghana and Malawi, provide insight into household valuation of SQ‐LNS, and how household characteristics, constraints, experience with SQ‐LNS, and other circumstances are associated with WTP.

Before summarizing and reflecting on the policy implications of this collection of results, there are a number of limitations to acknowledge. First, because respondents to the WTP surveys did not face an actual budget constraint, stated WTP is likely influenced by positive hypothetical bias (Whittington, 2010). In several settings, stated WTP for SQ‐LNS has been shown to be roughly double compared to estimates of revealed WTP, that is, WTP elicited under incentive‐compatible conditions in which money changed hands (Adams et al., 2016; Lybbert et al., 2016; Segrè et al., 2015). We therefore treat our estimates of stated WTP as an upper bound on true WTP. Conversely, it is conceivable that although respondents were asked to imagine that they were no longer receiving SQ‐LNS for free, receiving free product could have put downward pressure on WTP. This “anchoring” effect may have acted as a partial counterbalance to the inflationary effect of hypothetical bias (Fischer, Karlan, McConnell, & Raffler, 2014; Kőszegi & Rabin, 2006).

Second, differences in the framing of WTP elicitation in Ghana and Malawi may have artificially influenced the difference in stated WTP between the sites. First, respondents in Malawi were asked about their WTP for a week's supply, whereas in Ghana, they were asked about a day's supply. The implied WTP per day's supply in Malawi may have been higher had respondents in Malawi also been asked about WTP for a day's supply. Also, the randomized starting bids were substantially higher in Ghana than in Malawi. Although within‐site tests showed no statistically significant difference in WTP by randomized starting bid in either Ghana or Malawi, lower starting bids in Malawi may have led to lower WTP. The potential effect of both of these framing issues is a widening of the difference in WTP between the two sites, although they would not affect within‐site estimated relationships.

In the interest of protecting the scientific validity of the randomized trials and to avoid potentially misinforming respondents (because the efficacy results of the trials were not known when the WTP surveys were administered), respondents to the WTP surveys were not given any information about potential benefits (in either the short or long term) of consuming SQ‐LNS. Therefore, another limitation is that what we can claim about the role of experience in shaping WTP for SQ‐LNS is limited to experience accumulated in the very immediate term and does not account for how potential longer term benefits or costs, learned through experience, information, or social networks, may influence WTP. Nor are we able to make claims about the potential role of experience outside the context of a randomized controlled trial.

Finally, the extent to which our findings can be generalized is limited because our sample is composed of respondents from households in which a pregnant member sought out early (before 20 weeks of gestation) formal prenatal care, and households that do not seek early formal prenatal care may differ in their characteristics and valuation of a product such as SQ‐LNS.

Despite these limitations, the results presented here highlight several issues relevant to policymakers, government agencies, or aid organizations interested in forecasting potential demand for SQ‐LNS and designing strategies to deliver them. First, the contrasting results from Ghana and Malawi suggest that the level of demand for SQ‐LNS may be very context‐specific. In Ghana across the three periods (pregnancy, postpartum, and childhood), average WTP for a day's supply of SQ‐LNS ranged from $0.38 to $0.57, and long‐term WTP ranged from $0.22 to $0.46. In Malawi, WTP for a day's supply ranged from $0.11 to $0.22. To put these levels of WTP in context, in the Ghana sample, average daily per capita expenditures on food were approximately $1.38, whereas in Malawi, they were approximately $0.41 (unpublished data). Across the various rounds of WTP data collection, average WTP for a day's supply of SQ‐LNS, then, ranged from 28% to 41% of per capita food expenditures in Ghana and 27–54% of daily per capita food expenditures in Malawi. So while the average levels of WTP were substantially lower in Malawi than in Ghana, WTP relative to what households were spending on food was comparable in the two samples.

**Figure 1:**
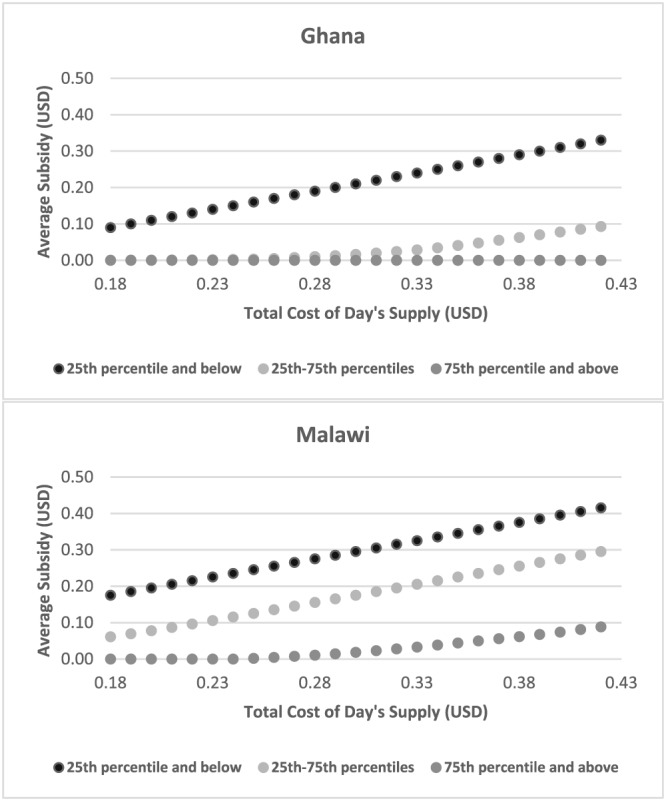
Average subsidies required to cover the total estimated cost of a day's supply of small‐quantity lipid‐based nutrient supplements by percentiles of willingness to pay

Producing SQ‐LNS at a new facility in Niger was estimated (volume‐weighted average over a 10‐year production cycle) to cost approximately $0.14 for a day's supply (iLiNS Project, 2015). Additional costs associated with transportation, distribution, marketing, etc. would vary widely depending on the modality of distribution; for example, home distribution would likely be more expensive than distribution from clinics. Moreover, it is not known how demand (and, ultimately, levels of consumption of SQ‐LNS) might vary across different distribution mechanisms. That said, assuming that additional costs might range from 30% to 200% of production costs (total costs for a day's supply ranging from $0.18–$0.42), Figure 1 shows the average size of the subsidy that would be required such that stated WTP plus the subsidy would cover the total cost of a day's supply of SQ‐LNS for households at different segments of the distribution of WTP. For households whose WTP was below the 25th percentile, average subsidies would be in the range of $0.09–$0.33 for Ghana (top panel) and $0.18–$0.42 for Malawi (bottom panel). For households in the Ghana sample whose WTP was between the 25th and 75th percentiles, subsidies would not be required until additional costs exceeded approximately 85% of SQ‐LNS production costs (total cost of $0.26/day's supply). In Malawi, average subsidies for these households would range from $0.06 to $0.30. Subsidization would not be required for households in Ghana whose WTP was at or above the 75th percentile even if additional costs reached 200% of the cost of production, whereas in Malawi subsidies would be required if total costs for a day's supply exceeded $0.26. These results suggest that for a sizeable portion of this particular population in Ghana—a predominantly nonfarming population earning their living hawking goods, running shops and kiosks, providing skills and services in a semi‐urban setting—there may be scope for passing on to consumers at least some of the costs associated with producing, marketing, and delivering SQ‐LNS. Among our sample in Malawi, drawn from a population of mainly subsistence farmers and fishers, substantial subsidies would be required for all but those at the top end of the distribution of WTP.

**Figure 2:**
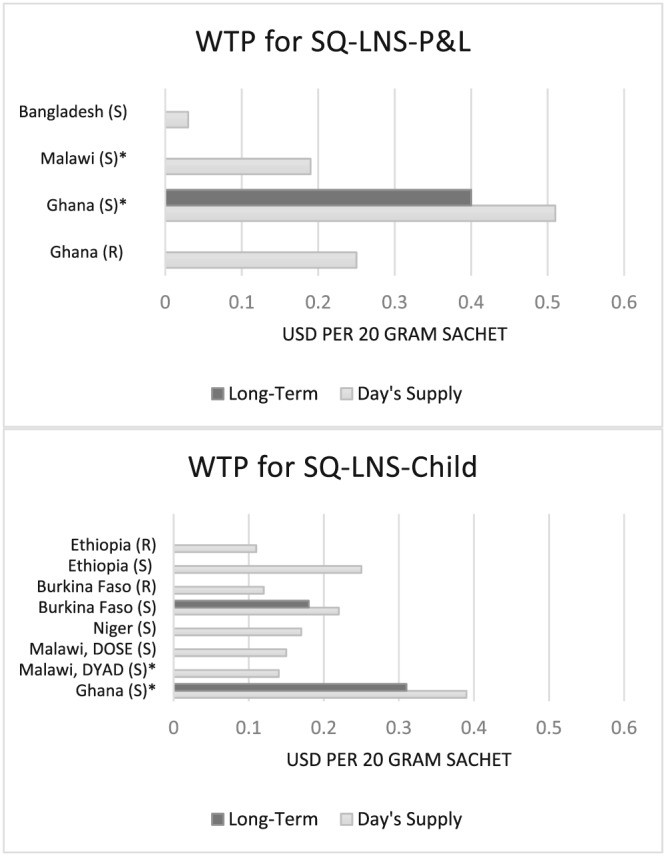
Willingness to pay for SQ‐LNS‐P&L and SQ‐LNS‐Child. SQ‐LNS‐P&L = small‐quantity lipid‐based nutrient supplements for pregnant and lactating women; SQ‐LNS‐Child = small‐quantity lipid‐based nutrient supplements for child consumption; S = stated; R = revealed (money changed hands in determining WTP); ^*^result from current study

Several recent studies have also looked directly at WTP for SQ‐LNS and provide relevant points of comparison for our estimates of WTP in Ghana and Malawi. Eliciting WTP using an experimental procedure in Ghana, Adams et al. (2016) found that mothers were willing to pay an average of $1.74 for a week's supply of SQ‐LNS‐P&L (or $0.25/day's supply). A recent effectiveness trial of SQ‐LNS in Bangladesh (Mridha et al., 2016) known as the Rang‐Din Nutrition Study also collected stated WTP for SQ‐LNS‐P&L. Average WTP for a day's supply of SQ‐LNS‐P&L among the Rang‐Din Nutrition Study population was approximately $0.03 (unpublished data). These average estimates of WTP for SQ‐LNS‐P&L are summarized alongside the estimates presented in this paper in the top panel of Figure 2.

An experimental auction in Burkina Faso revealed an average WTP for SQ‐LNS‐Child of $0.85 for a week's supply (or $0.12/day's supply; Lybbert et al., 2016). In another study, Segrè et al. (2015) elicited WTP for Nutributter®, an LNS product for children, from respondents in urban Ethiopia. That study found that average WTP for a week's supply of Nutributter® based on an experimental auction was approximately $0.80 (or $0.11/day's supply), and average stated WTP was over twice that amount. Tripp et al. (2011) also elicited stated WTP for Nutributter® and found that after a 2‐week trial period in which mothers were provided with Nutributter® to feed to their child, their average stated WTP for a day's supply was $0.22 and $0.11 in urban and rural Niger, respectively. Finally, two additional iLiNS studies, one in Malawi known as iLiNS‐DOSE, and one in Burkina Faso known as iLiNS‐ZINC also collected data on WTP for SQ‐LNS‐Child. Across several rounds of data collection, average stated WTP for a day's supply of SQ‐LNS‐Child was $0.15 in the Malawi DOSE site and $0.22 in Burkina Faso, and long‐term stated WTP for a day's supply of SQ‐LNS‐Child in Burkina Faso was $0.18 (unpublished authors' calculations). These WTP estimates for SQ‐LNS‐Child are summarized in the bottom panel of Figure 2.

Our results and the range of estimates of WTP from other studies suggest that although a hybrid delivery strategy that includes a market‐based mechanism might be feasible in settings such as semi‐urban Ghana, the proportion of households who might be expected to pay an out‐of‐pocket cost for SQ‐LNS would be much smaller in more resource‐constrained settings such as Malawi. In settings where policymakers expect SQ‐LNS to be effective and choose to promote them, private demand should be assessed in the local context, preferably using an incentive‐compatible mechanism that captures the persistence of demand over time (e.g., Lybbert et al., 2016). Where private demand is low, higher levels of public investment will be required to reach much of the target population of women and children.

Leveraging the randomized design of the iLiNS trials that provided SQ‐LNS to some households and not others, we found that in general, personal experience with SQ‐LNS did not influence WTP among the women who were consuming SQ‐LNS and, later, feeding them to their infants. Among household heads in Ghana, there was similarly no difference in WTP between the LNS and capsules groups. In some regards, this finding has positive implications for strategies to deliver the supplements. If there were unexpected nonmonetary costs associated with consuming SQ‐LNS (e.g., an unpleasant taste, unpleasant physical side effects, or intrahousehold tension over ensuring that SQ‐LNS was only consumed by target household members) that drove down WTP, then even if SQ‐LNS were provided for free, identifying mechanisms to encourage their consumption by vulnerable populations would be more challenging. Our results suggest that this is not the case, even after many months of consuming the product, as the downward trend in WTP over time did not differ by intervention group. Our results also suggest, though, that the benefits nutritionists and policymakers care about—benefits such as improvements in birth outcomes and linear growth in the case of Ghana—were either not observed by our survey respondents or were not directly valued by them. This may point to an opportunity for social marketing and/or information campaigns to provide information to households regarding the private benefits that may be linked to early childhood nutritional status, such as improvements in educational attainment and in economic productivity later in life. The downward trend in WTP over time suggests that marketing or promotional activities designed to stimulate and sustain demand would need to be ongoing over the course of recommended consumption, which would increase overall programme costs.

We found that in Ghana, WTP for SQ‐LNS for both maternal and child consumption was higher among heads of households than among mothers. In our sample, over 90% of heads of household indicated that they were primarily responsible for making purchase decisions related to food and for purchase decisions related to health care for their households. Only about a quarter of mothers indicated that they were primarily responsible for these purchase decisions in their household. The finding that WTP was lower among mothers, then, may be related to control over household resources in this setting and points to a potential need to include both mothers, who would be directly consuming SQ‐LNS or feeding them to their young children, and heads of household in, for example, social marketing efforts.

We also found that WTP for SQ‐LNS both for maternal consumption postpartum and for child consumption was, on average, higher for male children than female children in Ghana. In light of the lack of evidence of gender bias in nutritional outcomes in Ghana, this finding is a bit puzzling and deserves more research to uncover its underlying mechanism. At a minimum, though, policymakers should be aware of the potential for differential demand by child gender in Ghana.

In both Ghana and Malawi, we found a consistent negative association between household food insecurity and WTP for SQ‐LNS, even after controlling for household assets. This result is noteworthy because it is possible that women and young children in more food‐insecure households are also those for whom SQ‐LNS may help protect against critical nutrient gaps in their diets, as was found in Bangladesh (Mridha et al., 2016). This result suggests that if a cost‐effective mechanism for targeting based on household food insecurity could be identified, it might be used to encourage SQ‐LNS consumption in food‐insecure households.

The challenges associated with delivering a regular‐use product such as SQ‐LNS to target members of nutritionally vulnerable households are many. SQ‐LNS is an unfamiliar product. Many of the hoped‐for benefits may be difficult for households to directly observe and are long term in nature. Households have to make trade‐offs in where they choose to allocate their scarce resources. Should SQ‐LNS become available (either via markets, through health clinics, or by some other delivery mechanism), demand will be low for some stakeholder groups but may be higher among others. In contexts were SQ‐LNS are likely to be effective, and if policymakers choose to promote them, possible delivery mechanisms for SQ‐LNS will need to be carefully considered within the specific context, and education/information dissemination and targeted subsidies will likely be part of any distribution strategy.

## CONFLICTS OF INTEREST

The authors declare that they have no conflicts of interest.

## CONTRIBUTIONS

KPA and SAV designed the contingent valuation studies. EA and TP assisted in the study designs and supervised data collection. KPA, SAV, and KGD conceptualized the analyses. SAA, KM, SAV, UA, and KGD designed and supervised the iLiNS trials. MA managed the iLiNS trials. KPA analysed and interpreted the data. KPA drafted the manuscript, and all authors critically commented on drafts and approved the final manuscript.

## Supporting information

Table A1. Nutrient Composition of iLiNS DYAD Supplements
Table A2. Definition of Covariates
Table A3. Morbidity Reference Periods
Table A4. Ghana Average Value of Covariates by Round
Table A5. Malawi Average Value of Covariates by Round
Table A6. Effect of LNS Group on WTP
Table A7. Factors Associated with WTP during the Pregnancy Period, Continuous Morbidity Variables
Table A8. Factors Associated with WTP during the Postpartum Period, Continuous Morbidity Variables
Table A9. Factors Associated with WTP during the Child Period, Continuous Morbidity Variables
Table A10. Factors Associated with WTP during the Pregnancy Period, 30‐day Reference Period
Table A11. Factors Associated with WTP during the Postpartum Period, 30‐day Reference Period
Table A12. Factors Associated with WTP during the Child Period, 30‐day Reference Period
Figure A1. Timeline of hWTP Data Collection
Figure A2. Sample Bidding Tree
Figure A3. Average WTP with 95% Confidence Intervals by Period/Round and Intervention Group
Figure A4. Ghana Pregnancy Period Adjusted Predicted WTP with 95% Confidence Intervals
Figure A5. Ghana Postpartum Period Adjusted Predicted WTP with 95% Confidence Intervals
Figure A6. Ghana Child Period Adjusted Predicted WTP with 95% Confidence Intervals
