# Supplementary material for: Willingness to pay for small‐quantity lipid‐based nutrient supplements for women and children: Evidence from Ghana and Malawi
Source: Matern Child Nutr. 2017 Sep 28;14(2):e12518. doi: 10.1111/mcn.12518 (PMC6088232; doi:10.1111/mcn.12518)
Supplement: Supplementary file 1 — Table A1. Nutrient Composition of iLiNS DYAD Supplements Table A2. Definition of Covariates Table A3. Morbidity Reference Periods Table A4. Ghana Average Value of Covariates by Round Table A5. Malawi Average Value of Covariates by Round Table A6. Effect of LNS Group on WTP Table A7. Factors Associated with WTP during the Pregnancy Period, Continuous Morbidity Variables Table A8. Factors Associated with WTP during the Postpartum Period, Continuous Morbidity Variables Table A9. Factors Associated with WTP during the Child Period, Continuous Morbidity Variables Table A10. Factors Associated with WTP during the Pregnancy Period, 30‐day Reference Period Table A11. Factors Associated with WTP during the Postpartum Period, 30‐day Reference Period Table A12. Factors Associated with WTP during the Child Period, 30‐day Reference Period Figure A1. Timeline of hWTP Data Collection Figure A2. Sample Bidding Tree Figure A3. Average WTP with 95% Confidence Intervals by Period/Round and Intervention Group Figure A4. Ghana Pregnancy Period Adjusted Predicted WTP with 95% Confidence Intervals Figure A5. Ghana Postpartum Period Adjusted Predicted WTP with 95% Confidence Intervals Figure A6. Ghana Child Period Adjusted Predicted WTP with 95% Confidence Intervals [file MCN-14-e12518-s001.docx]

Supporting Information to:

Willingness-to-Pay for Small-Quantity Lipid-Based Nutrient Supplements for Women and Children:

Evidence from Ghana and Malawi

**1. Nutrient Content**

Table A1 summarizes the nutrient content of the SQ-LNS products and capsules used in the randomized controlled iLiNS DYAD trials in Ghana and Malawi.

Table A1. Nutrient Composition of iLiNS DYAD Supplements

|  | Nutrient Content per Daily Ration | | | |
| --- | --- | --- | --- | --- |
| Nutrient | LNS-Child | LNS-P&L | Multiple Micronutrient Capsule | Iron-Folic Acid Capsule |
| Daily Ration (g/day) | 20 | 20 |  |  |
| Total energy (kcal) | 118 | 118 |  |  |
| Protein (g) | 2.6 | 2.6 |  |  |
| Fat (g) | 9.6 | 10 |  |  |
| Linoleic acid (g) | 4.46 | 4.59 |  |  |
| α-Linoleic acid (g) | 0.58 | 0.59 |  |  |
| Vitamin A (µg RE) | 400 | 800 | 800 |  |
| Vitamin C (mg) | 30 | 100 | 100 |  |
| Vitamin B_1_ (mg) | 0.3 | 2.8 | 2.8 |  |
| Vitamin B_2_ (mg) | 0.4 | 2.8 | 2.8 |  |
| Niacin (mg) | 4 | 36 | 36 |  |
| Folic acid (mg) | 80 | 400 | 400 | 400 |
| Pantothenic acid (mg) | 1.8 | 7 | 7 |  |
| Vitamin B_6_ (mg) | 0.3 | 3.8 | 3.8 |  |
| Vitamin B_12_ (µg) | 0.5 | 5.2 | 5.2 |  |
| Vitamin D (IU) | 200 | 400 | 400 |  |
| Vitamin E (mg) | 6 | 20 | 20 |  |
| Vitamin K (µg) | 30 | 45 | 45 |  |
| Iron (mg) | 6 | 20 | 20 | 60 |
| Zinc (mg) | 8 | 30 | 30 |  |
| Cu (mg) | 0.34 | 4 | 4 |  |
| Calcium (mg) | 280 | 280 |  |  |
| Phosphorus (mg) | 190 | 190 |  |  |
| Potassium (mg) | 200 | 200 |  |  |
| Magnesium (mg) | 40 | 65 |  |  |
| Selenium (µg) | 20 | 130 | 130 |  |
| Iodine (µg) | 90 | 250 | 250 |  |
| Manganese (mg) | 1.2 | 2.6 | 2.6 |  |

Sources: Adu-Afarwuah et al. (2015); Adu-Afarwuah et al. (2016)

**2. Timeline**

The timing of WTP data collection by period and by trial for a representative participant is shown in Figure A1. WTP data were collected twice during the pregnancy period, once during the postpartum period, and twice during the child period.


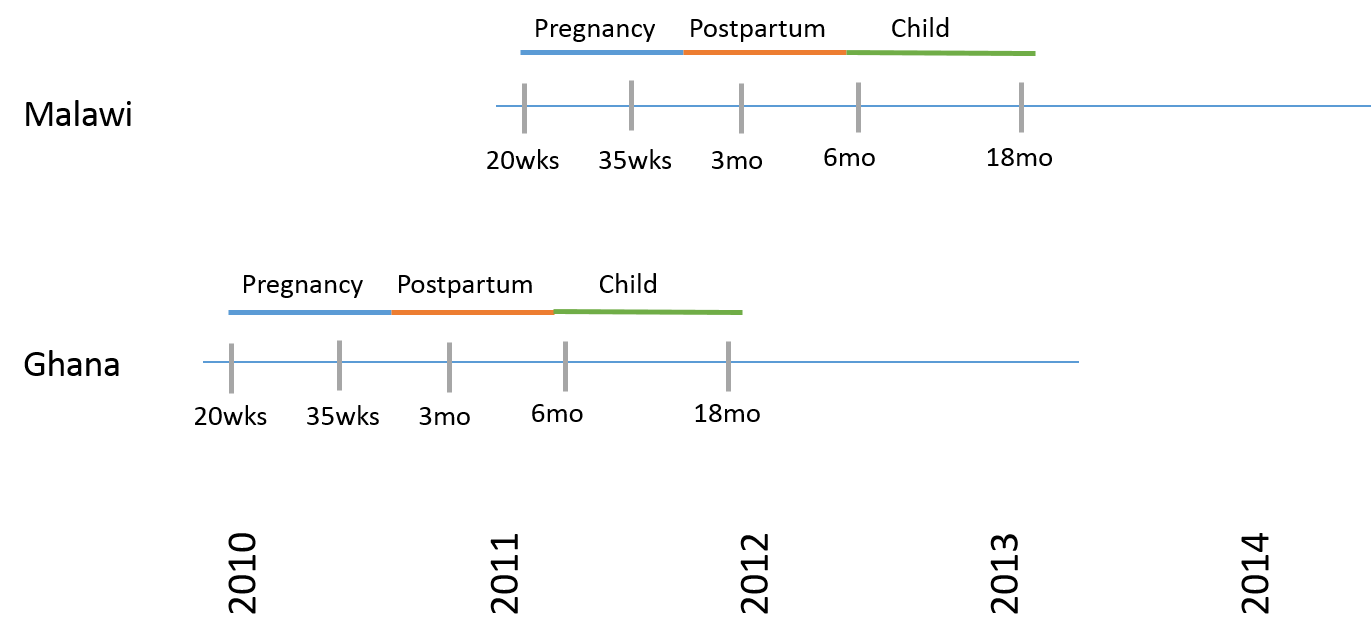


Figure A1. Timeline of hWTP Data Collection

**3. Description of the Contingent Valuation Survey**

The contingent valuation survey began with a short statement about undernutrition and nutrient supplements, in general, as a means through which the nutrient content of the diets of mothers and young children can be enhanced. The text of this introductory information is detailed below. Respondents were then shown a sample of the SQ-LNS product, read a brief description of the product, and then asked to consider how much the product might be worth to them if it was available for sale at a nearby kiosk/stall. Because some respondents (or someone in the respondent’s household in the case of heads of household) were receiving SQ-LNS for free as part of the randomized trial, all respondents were asked to imagine a scenario in which the iLiNS study had run out of money or was ending so the trial (and therefore free provision of SQ-LNS) was ending that day. Respondents were also reminded to think about their household’s daily expenditures and what they could afford based on their household’s income.

Using a bidding tree structure, an example of which is shown in Figure A2, respondents were first asked to indicate whether they would be willing to pay anything for day’s supply (20 grams)^[[1]](#footnote-1)^ of SQ-LNS. If the respondent indicated s/he was willing to pay at least something for SQ-LNS, s/he was then led through the bidding tree of prices, with the price increasing or decreasing depending on the response to a dichotomous choice question at each node in the tree.^^[[2]](#footnote-2)^^ Once an end node in the tree was reached, the respondent was asked to state his/her maximum WTP. To control for starting point bias, the starting price was randomized across respondents.^[[3]](#footnote-3)^

Given that SQ-LNS are meant to be consumed daily for many months, after respondents reported their maximum WTP for a day’s supply, they were asked two additional questions meant to induce thought about their WTP for the product throughout the relevant time period (i.e., throughout pregnancy or throughout the first six months postpartum for LNS-P&L and from 6-18 months of age for LNS-Child) rather than a single day’s supply.^[[4]](#footnote-4)^ In Ghana, the stated maximum WTP for a day’s supply and stated long-term WTP throughout the period provide our estimates of valuation of the SQ-LNS products.^[[5]](#footnote-5)^ An error in the printing of the WTP surveys in Malawi rendered the estimates of long-term WTP unreliable, so the Malawi analysis is limited to WTP for a day’s supply.


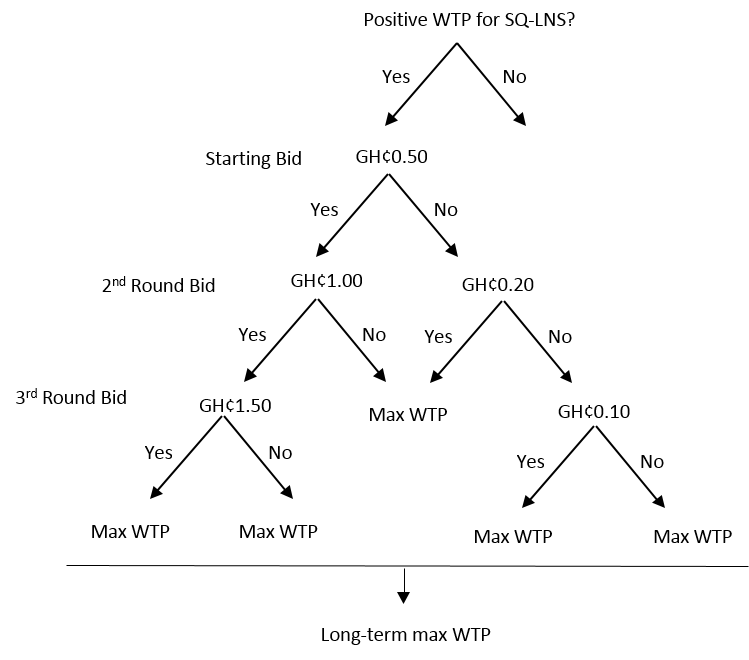


Figure A2. Sample Bidding Tree

*3.1 Introductory Information and Preamble to WTP Elicitation*

During their participation in the contingent valuation surveys, respondents were read pre-scripted information to introduce them to the WTP study and provide basic information on SQ-LNS. Below we provide samples of this text for the pregnancy period in Malawi and the child period in Ghana.

*3.1.1 Malawi*

Introductory information read to participants when eliciting WTP during the pregnancy period:

As you may know, undernutrition in young children is a problem in Malawi. Children can become undernourished when the foods they eat do not provide them with enough vitamins, minerals, and energy to grow properly. Undernutrition can even begin during pregnancy before the child is born if a pregnant mother does not get sufficient amounts of vitamins and minerals, which can affect the health of both the mother and her baby.

The nutrients contained in the staple foods in the Malawian diet, such as cassava and maize, are often not sufficient to ensure that a mother and the baby she is carrying are properly nourished during pregnancy. There are a variety of nutritional supplements that pregnant women might use to add to the nutrients in their normal diet. Show the respondent the tablets, Bonya, and chiponde chatsopano. The Plumpy Nut product that you may have heard about or used is designed to *treat* *severe* undernutrition, please remember that the new Chiponde chatsopano is meant to *prevent* undernutrition.

I want to focus on the Chiponde chatsopano and Bonya today. After I describe each of these nutritional supplements, I will ask you about how much you would be willing to pay to purchase the nutritional supplement for [yourself during pregnancy]/[pregnant members of your household].

During the time I am talking with you, I would like you to pretend that the money for the Chiponde chatsopano study ran out so the Chiponde chatsopano study is ending today. This would mean that the study would not be making deliveries of any supplements or tablets. Of course this is not true, but for the rest of the time I am talking with you and asking you questions, I want you to think about what I am saying and answer my questions as though the Chiponde chatsopano study has stopped.

Information about SQ-LNS (local name Chiponde Chatsopano) read to participants when eliciting WTP during the pregnancy period:

Now, I would like to talk to you about your willingness to pay for Chiponde chatsopano. Chiponde chatsopano is a new, groundnut-based vitamin and mineral supplement, and it is one way to provide pregnant women with extra vitamins and minerals during pregnancy. Pregnant women should take 1 sachet per day mixed with other foods. It should be taken every day throughout pregnancy.

Now, I want you to imagine that a new stall is built within easy walking distance of your house, and you find out that the kiosk owner is selling Chiponde chatsopano. I am going to ask you some questions about how much you would be willing to pay the new kiosk owner to purchase one week’s supply (7 days) of Chiponde chatsopano [Show the respondent the Chiponde chatsopano sachet], which is 20 grams.

It is important that you give me an honest answer. There is no right or wrong answer; we are only interested in learning about how much you would be willing to pay for Chiponde chatsopano. When you think about your answer, remember your household’s daily expenses and what you can afford based on your household’s income. If you are willing to pay for Chiponde chatsopano then you would no longer have that money to spend on other things. Also, remember that you should be pretending the iLiNS study has stopped.

*3.1.2 Ghana*

Introductory information read to participants when eliciting WTP during the child period:

As you may know, undernutrition in young children is a problem in Ghana. Children can become undernourished when the foods they eat do not provide them with enough vitamins, minerals, and energy to grow properly.

The nutrients contained in the normal diet for young Ghanaian children, such a koko, weanimix/tombrown, mpotompoto/mashed yams, wakye, rice, banku, fufu etc. are often not sufficient to ensure that a young child is properly nourished. There are a variety of nutritional supplements that might be given to young children to add to the nutrients in their normal diet. SHOW THE RESPONDENT THE SOYA BEANS FLOUR, AND NKATEPA JUNIOR. I would like to talk to you about these two supplements today. After I describe each of these nutritional supplements, I will ask you about how much you would be willing to pay to purchase the nutritional supplement for the iLiNS child in your household.

During the time I am talking with you, I would like you to pretend that the money for the iLiNS study ran out so the iLiNS study is ending today. Of course this is not true, but for the rest of the time I am talking with you and asking you questions, I want you to think about what I am saying and answer my questions as though the iLiNS study has stopped. Do you have any questions before we begin?

Information about SQ-LNS (local name nkatepa junior) read to participants when eliciting WTP during the child period:

Nkatepa junior is a new, groundnut-based vitamin and mineral supplement, and it is one way to provide young children with extra vitamins and minerals when they are developing. Young children should be given 2 sachets per day mixed with other foods. Nkatepa junior should be given to young children every day when they are between the ages of 6 months and approximately 18 months.

Now, I want you to imagine that a new kiosk is built within walking distance of your house, and you find out that the kiosk owner is selling nkatepa junior. I am going to ask you some questions about how much you would be willing to pay the new kiosk owner to purchase 2 sachets of nkatepa junior [SHOW THE RESPONDENT 2 NKATEPA SACHETS], each containing 10 grams.

It is important that you give me an honest answer. There is no right or wrong answer, we are only interested in learning about how much you would be willing to pay for nkatepa junior. When you think about your answer, remember your household’s daily expenses and what you can afford based on your household’s income. If you are willing to pay for nkatepa junior then you would no longer have that money to spend on other things. Also, remember that you should be pretending the iLiNS study has stopped.

**4. Covariates**

Household demographic and socioeconomic data were collected at or shortly after maternal enrollment into the randomized trials. These data included information on household composition, education, household asset ownership, and household food insecurity. Infant anthropometrics (e.g., weight and length) were measured at birth and again several times after birth and up to 18 months of age. Maternal and child morbidity data were collected biweekly for mothers and weekly for infants through the entirety of the mother’s and infant’s participation in the trial. The covariates used in our regression analysis are defined in Table A2.

Table A2. Definition of Covariates

| Variable | Definition |
| --- | --- |
| LNS Group (0/1) | Dichotomous variable = 1 if mother/infant DYAD was randomized to receive SQ-LNS throughout the trial. |
| Months from Enrollment | Months from enrollment into trial to WTP survey administration. |
| Months from Birth | Months from birth of infant to WTP survey administration. |
| Lean Season (0/1) | Dichotomous variable = 1 if WTP survey was administered during the lean season |
| Mangochi (0/1) | Dichotomous variable = 1 if the site of enrollment (Malawi trial) was Mangochi |
| Mother (0/1) | = 1 if respondent to WTP survey is mother (= 0 if head of household)^[[6]](#footnote-6)^ |
| Respondent Age | Respondent’s age in years. |
| Respondent Education | Respondent’s years of education. |
| Asset Index | Proxy for household socioeconomic status. |
| HFIAS Score | Household food insecurity access scale score closest to WTP survey administration. |
| Primiparity | Dichotomous variable = 1 if infant is mother’s first child. |
| Maternal Poor Appetite (0/1) | Dichotomous variable = 1 if any days of reported maternal poor appetite during reference period.* |
| Maternal Nausea or Vomiting (0/1) | Dichotomous variable = 1 if any days of reported maternal nausea and/or vomiting during reference period. |
| Maternal Diarrhea (0/1) | Dichotomous variable = 1 if any days of reported maternal diarrhea during reference period. |
| Infant Ill (0/1) | Dichotomous variable = 1 if any days of reported infant general illness during reference period. |
| Infant Poor Appetite (0/1) | Dichotomous variable = 1 if any reported infant poor appetite during reference period. |
| Infant Vomiting (0/1) | Dichotomous variable = 1 if any reported infant vomiting during reference period. |
| Infant Diarrhea (0/1) | Dichotomous variable = 1 if any reported infant diarrhea during reference period. |
| Infant Male (0/1) | Dichotomous variable = 1 if infant is male. |
| BMIZ | Infant’s body mass index for age z-score at birth. |
| LAZ | Infant’s length-for-age z-score at birth/measurement closest to WTP survey administration. |
| WLZ | Infant’s weight-for-length z-score at the measurement closest to WTP survey administration. |

*Reference periods are defined in Table A3.

Table A3. Morbidity Reference Periods

| **Period and Round** | **Definition** |
| --- | --- |
| Pregnancy 1 | Maternal enrollment into trial through date of WTP round 1 |
| Pregnancy 2 | Maternal enrollment into trial through date of WTP round 2 |
| Postpartum | Birth of infant through date of WTP round 3 |
| Child 1 | Infant six months old through date of WTP round 4 |
| Child 2 | Infant six months old through date of WTP round 5 |

For Ghana and Malawi, respectively, Tables A4 and A5 show the mean value of all covariates used in the regression analysis to estimate the factors associated with WTP in each period.

Table A4. Ghana Average Value of Covariates by Round

|  | Pregnancy | | Postpartum | Child | |
| --- | --- | --- | --- | --- | --- |
| Variable | 1 | 2 | 1 | 1 | 2 |
| LNS Group (0/1) | 0.33 | 0.36 | 0.33 | 0.34 | 0.34 |
| Months from Enrollment | 1.6 | 4.6 |  |  |  |
| Months from Birth |  |  | 3.6 | 7.3 | 18.7 |
| Lean Season (0/1) | 0.43 | 0.45 | 0.45 | 0.35 | 0.39 |
| Mother (0/1) | 0.58 | 0.60 | 0.57 | 0.60 | 0.58 |
| Respondent Age | 31.5 | 31.5 | 31.9 | 31.4 | 31.4 |
| Respondent Education (y) | 8.0 | 8.0 | 7.9 | 7.8 | 7.9 |
| Asset Index | 0.04 | -0.01 | 0 | 0.14 | 0.03 |
| HFIAS Score | 2.43 | 1.48 | 1.54 | 1.77 | 1.77 |
| Primiparity (0/1) | 0.34 | 0.33 | 0.35 | 0.30 | 0.31 |
| Maternal Poor Appetite (0/1) | 0.28 | 0.47 | 0.15 |  |  |
| Maternal Nausea or Vomiting (0/1) | 0.52 | 0.70 |  |  |  |
| Maternal Diarrhea (0/1) | 0.20 | 0.41 | 0.10 |  |  |
| Infant Ill (0/1) |  |  | 0.31 | 0.28 | 0.93 |
| Infant Poor Appetite (0/1) |  |  | 0.15 | 0.22 | 0.89 |
| Infant Vomiting (0/1) |  |  | 0.28 | 0.27 | 0.82 |
| Infant Diarrhea (0/1) |  |  | 0.17 | 0.13 | 0.65 |
| Infant Male (0/1) |  |  | 0.50 | 0.46 | 0.50 |
| BMIZ at Birth |  |  | -0.62 |  |  |
| LAZ^1^ |  |  | -0.65 | -0.80 | -0.85 |
| WLZ^2^ |  |  |  | -0.04 | -0.55 |
| N | 541 | 430 | 526 | 297 | 435 |

^1^In the postpartum period, LAZ is as measured at birth. In the child periods, LAZ is at the measurement closest to WTP survey administration.

^2^WLZ is at the measurement closest to WTP survey administration.

Table A5. Malawi Average Value of Covariates by Round

|  | Pregnancy | | Postpartum | Child | |
| --- | --- | --- | --- | --- | --- |
| Variable | 1 | 2 | 1 | 1 | 2 |
| LNS Group (0/1) | 0.33 | 0.35 | 0.35 | 0.32 | 0.34 |
| Months from Enrollment | 1.2 | 4.2 |  |  |  |
| Months from Birth |  |  | 3.2 | 7.2 | 18.1 |
| Lean Season (0/1) | 0.38 | 0.47 | 0.47 | 0.33 | 0.46 |
| Mangochi (0/1) | 0.33 | 0.28 | 0.24 | 0.30 | 0.45 |
| Respondent Age | 24.7 | 24.2 | 25.0 | 25.0 | 24.8 |
| Respondent Education (y) | 3.9 | 3.9 | 3.6 | 4.0 | 4.2 |
| Asset Index | 0.05 | -0.04 | -0.08 | -0.03 | 0.10 |
| HFIAS Score | 5.01 | 5.06 | 5.19 | 4.04 | 3.27 |
| Primiparity (0/1) | 0.21 | 0.26 | 0.19 | 0.21 | 0.18 |
| Maternal Poor Appetite (0/1) | 0.31 | 0.47 | 0.07 |  |  |
| Maternal Nausea or Vomiting (0/1) | 0.35 | 0.54 |  |  |  |
| Maternal Diarrhea (0/1) | 0.15 | 0.32 | 0.14 |  |  |
| Infant Ill (0/1) |  |  | 0.59 | 0.32 | 0.84 |
| Infant Poor Appetite (0/1) |  |  | 0.28 | 0.20 | 0.77 |
| Infant Vomiting (0/1) |  |  | 0.34 | 0.25 | 0.91 |
| Infant Diarrhea (0/1) |  |  | 0.28 | 0.15 | 0.68 |
| Infant Male (0/1) |  |  | 0.45 | 0.47 | 0.46 |
| BMIZ at Birth |  |  | -0.05 |  |  |
| LAZ^*^ |  |  | -1.02 | -1.27 | -1.62 |
| WLZ^*^ |  |  |  | 0.40 | -0.17 |
| N | 506 | 374 | 337 | 413 | 290 |

^*^Notes: In the postpartum period, LAZ is as measured at birth. In the child periods, LAZ is at the measurement closest to WTP survey administration. WLZ (child period) is at the measurement closest to WTP survey administration.

**5. Effect of LNS Group on WTP**


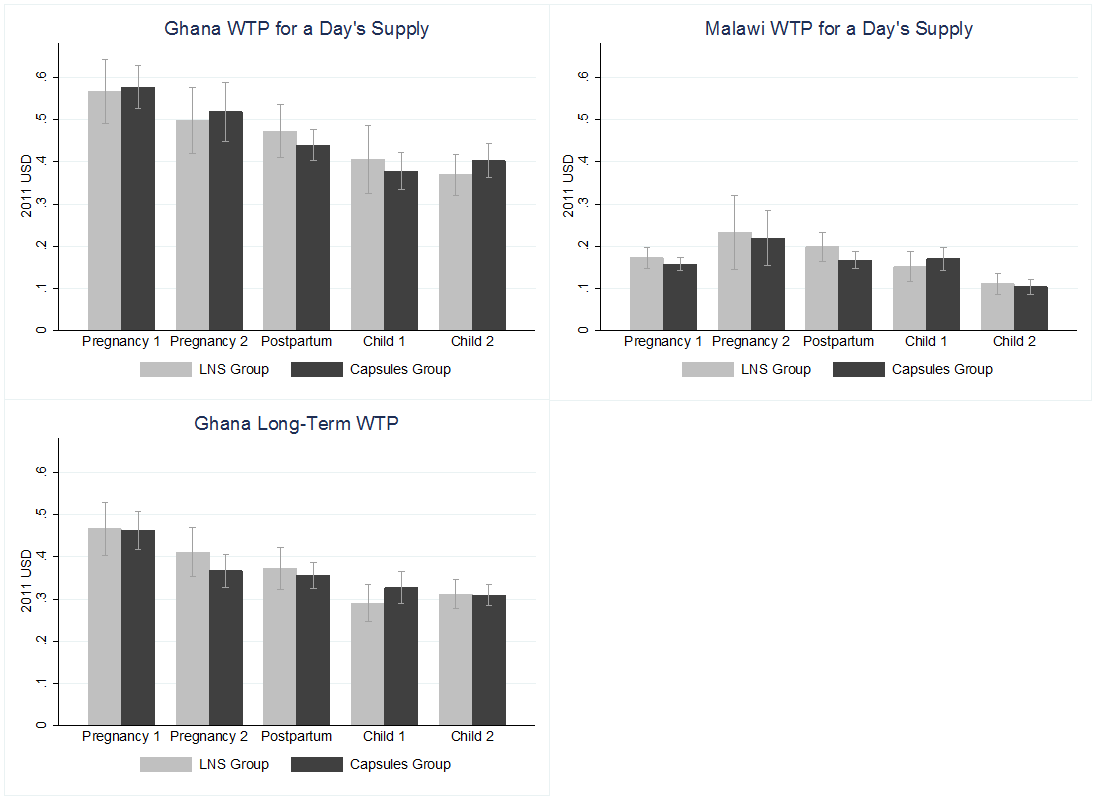


Figure A3. Average WTP with 95% Confidence Intervals by Period/Round and Intervention Group

Table A6. Effect of LNS Group on WTP

|  |  |  | Ghana | | | Malawi | | |
| --- | --- | --- | --- | --- | --- | --- | --- | --- |
|  |  |  | **Pregnancy**  **(1)** | **Postpartum**  **(2)** | **Child**  **(3)** | **Pregnancy**  **(4)** | **Postpartum**  **(5)** | **Child**  **(6)** |
| Day's Supply |  | LNS Group | -0.007 | 0.039 | 0.002 | 0.017 | 0.037 | -0.007 |
|  |  |  | (0.044) | (0.038) | (0.025) | (0.034) | (0.048) | (0.022) |
|  |  | Constant | 0.529*** | 0.237** | 0.413*** | 0.083** | 0.282*** | 0.204*** |
|  |  |  | (0.059) | (0.096) | (0.053) | (0.039) | (0.065) | (0.025) |
|  |  | N | 1019 | 538 | 750 | 961 | 376 | 747 |
|  |  | Wald Chi^2^ | 4.795 |  | 13.156 | 6.410 |  | 24.897 |
|  |  | Pseudo R^2^ |  | 0.014 |  |  | 0.242 |  |
| Long-Term Throughout Period |  | LNS Group | 0.024 | 0.021 | -0.006 |  |  |  |
|  |  |  | (0.032) | (0.031) | (0.018) |  |  |  |
|  |  | Constant | 0.436*** | 0.260*** | 0.356*** |  |  |  |
|  |  |  | (0.044) | (0.087) | (0.038) |  |  |  |
|  |  | N | 1014 | 538 | 749 |  |  |  |
|  |  | Wald Chi^2^ | 25.607 |  | 10.880 |  |  |  |
|  |  | Pseudo R^2^ |  | 0.006 |  |  |  |  |

Significance codes: *** (p < .01), ** (p < .05), * (p < .1).

Notes: Dependent variables stated WTP in 2011 US dollars. Controls for months from enrollment to WTP survey administration and indicators for randomized starting bid are included in all models (unreported). Standard errors, in parentheses, obtained via 50 bootstrap replications for pregnancy and child periods. Robust standard errors in parentheses for postpartum period.

**6. Heterogeneity**

The results in the main paper impose the assumption that the magnitude and direction of the estimated associations were, on average, the same across the sample. It is possible that the effect of group may have changed over time as women gained more experience with SQ-LNS and had time to experience any costs and short-term benefits associated with consuming it/feeding it to their infant. We therefore tested for heterogeneity in the effect of assignment to the LNS group over time. In Ghana, the household respondent to the WTP survey was randomly assigned as either the mother participating in the trial or her head of household. Because the effect of assignment to the LNS group may have been different for mothers who were themselves consuming SQ-LNS and, later, feeding it to their children compared to heads of household, we also tested for heterogeneity in the effect of LNS group on WTP by survey respondent. And finally, given heterogeneity in the effect of SQ-LNS on birth outcomes in Ghana by maternal parity (Adu-Afarwuah et al., 2015), we also assessed heterogeneity in the effect of randomization into the LNS group on WTP by maternal parity in Ghana. Heterogeneity in the effect of being in the LNS group on WTP over time was assessed by interacting intervention group with the continuous variable ‘months from enrollment’ in the pregnancy period and by interacting intervention group with the continuous variable ‘months from birth’ in the postpartum and child periods. Heterogeneity by survey respondent and by maternal parity were assessed through interactions between the indicator for intervention group and the dichotomous variables survey respondent or maternal parity.

In theory, experience consuming SQ-LNS, even in the short-term, could play an important role in a household’s willingness to pay for it, since with experience comes household knowledge of the associated private costs and short-term benefits. The results in Table A6 however, suggest that in the context of the randomized trials in both Ghana and Malawi, personal experience using SQ-LNS did not, in general, influence WTP. On the one hand, there did not appear to be substantial private short-term benefits^[[7]](#footnote-7)^ to consuming SQ-LNS that households in the LNS group were experiencing and reflecting in their valuation of the product. This held true over the course of each period, as there was no difference in the effect of LNS group on WTP over time in Ghana or Malawi (results unreported). It also held true among the primiparous mothers in Ghana for whom the clinical effect of SQ-LNS on some birth outcomes (i.e., weight, length, and head circumference) was particularly evident to researchers, though not necessarily the mothers themselves (results unreported).

On the other hand, there did not appear to be substantial non-monetary costs associated with consuming SQ-LNS that became apparent to households in the LNS group that subsequently reduced their valuation of the product. Even among the mothers who were consuming SQ-LNS and feeding it to their infants and therefore likely to bear the majority of any non-monetary costs, there was no difference in the effect of being in the LNS group on WTP between mothers and heads of household in Ghana (results unreported). This lack of an ‘experience’ effect on WTP is important given the intensity with which mothers and children were asked to consume SQ-LNS (daily for many months).

We also used interaction terms to assess heterogeneity in the relationship between WTP and the factors predicted to be associated with WTP. For each covariate in Table 2 of the main paper, heterogeneity in the association with WTP by intervention group and by WTP respondent was assessed using separate regressions, and the statistically significant interactions are shown in Figure A4. Among all of the pregnancy period covariates, in both Ghana and Malawi, there were no differences between intervention groups in the estimated association (that is, the interaction between each covariate and ‘LNS group’ was not statistically significant for any of the covariates), implying that the nature of the factors associated with WTP did not vary depending on personal experience with SQ-LNS in that period. However, in Ghana the estimated associations between WTP for a day’s supply of SQ-LNS and two factors, respondent education and maternal nausea or vomiting, were found to depend on who in the household responded to the WTP survey. If the survey respondent was the head of household, each additional year of education was associated with an approximate $0.02 increase in WTP. For mothers, however, the association was not statistically significant (top panel of Figure A4).

The relationship between WTP for a day’s supply of SQ-LNS and maternal nausea or vomiting during the pregnancy period also varied by survey respondent. Among heads of household, WTP was, on average, approximately $0.17 lower (p<.01) if the mother experienced nausear or vomiting than if the mother did not. When the WTP survey respondent was the mother, however, there was no differnce in WTP for a day’s supply of SQ-LNS among mothers who experienced nausea or vomiting during their pregnancy compared to those who did not. The adjusted predicted values of WTP by survey respondent and by maternal nausea or voimting are shown in the bottom panel of Figure A4.

|  |
| --- |
| 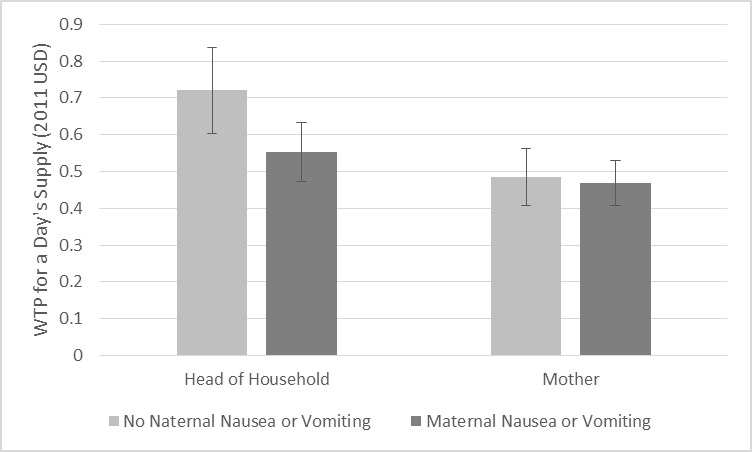 |

Figure A4. Ghana Pregnancy Period Adjusted Predicted WTP with 95% Confidence Intervals

For the postpartum period, we again tested for heterogeneity in these associations by intervention group and by survey respondent. Statistically significant interactions are shown in Figure A5.

In the postpartum period, the association between the lean season and both WTP for a day’s supply of SQ-LNS and long-term WTP was positive for heads of household, indicating that heads of household were, on average, willing to pay more for SQ-LNS (approximately $0.13 more for a day’s supply and $0.12 more in the long-term) in the lean season than in the harvest season. If the survey respondent was the mother, however, the association between the lean season and WTP was negative and not statistically significant. The adjusted predicted values of long-term WTP in the top left panel of Figure A5 show the statistically significantly higher average WTP in the lean season for heads of household but no seasonal difference in WTP among mothers. Also in the postpartum period, the association between respondent age and both WTP for a day’s supply and in the long-term was negative for mothers but positive for heads of household, though the size of the association was small in both cases, as demonstrated in the bottom left panel of Figure A5. Maternal poor appetite in the postpartum period was associated with a lower long-term WTP, all else constant, among heads of household, but there was no statistically significant difference in WTP among mothers who experienced poor appetite compared to those who did not (see the top right panel of Figure A5). Similarly, WTP for a day’s supply of SQ-LNS as well as long-term WTP was higher among heads of household if the infant experienced diarrhea during the period, but the association between infant diarrhea and WTP was not statistically significant among mothers (see middle left panel of Figure A5). There was also heterogeneity by survey respondent in the association between long-term WTP and the infant’s length-for-age z-score at birth. Shown graphically in the bottom right panel of Figure A5, the infant’s LAZ at birth was not associated with WTP among mothers, but there was a negative (albeit very small in magnitude) association between LAZ and long-term WTP among heads of household.

Finally, the association between maternal diarrhea in the postpartum period and both WTP for a day’s supply and long-term WTP for SQ-LNS were modified by intervention group. As shown in the middle right panel of Figure A5, in the LNS group the predicted adjusted long-term WTP was almost $0.32 higher if the mother experienced diarrhea than if she did not. In the non-LNS group (the combined capsule groups), maternal diarrhea was not statistically significantly associated with WTP. A caveat to this result is that the number of mothers who experienced diarrhea during the postpartum period was small.

In the postpartum period in Malawi, the intervention group did not modify any of the estimated associations.

| 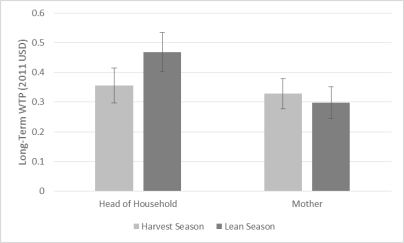 | 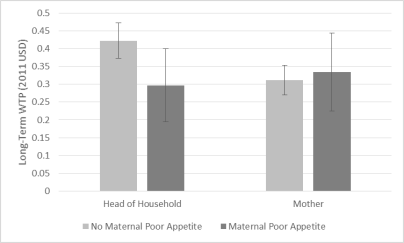 |
| --- | --- |
| 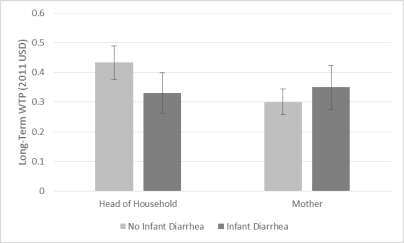 | 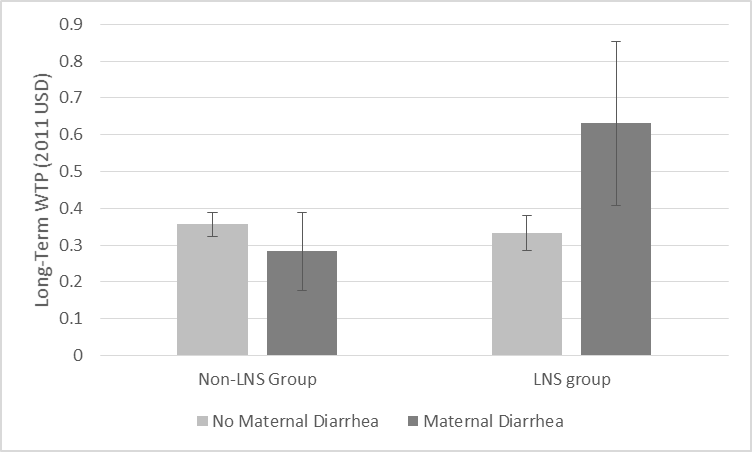 |
|  |  |

Figure A5. Ghana Postpartum Period Adjusted Predicted WTP with 95% Confidence Intervals

As in the other two periods, we also tested for heterogeneity in the estimated associations in the child period by intervention group for both Ghana and Malawi and by survey respondent for Ghana. Statistically significant interactions are shown in Figure A6. In Ghana, the association between respondent education and WTP for a day’s supply of SQ-LNS as well as long-term WTP was positive and statistically significant for heads of household but negative (though not statistically significant) for mothers.

Figure A6. Ghana Child Period Adjusted Predicted WTP with 95% Confidence Intervals

**6. Sensitivity Analysis**

In defining our covariates, we had to make a series of assumptions. In what follows, we describe the results of sensitivity analyses to test the robustness of our results to several key assumptions.

First, the maternal and child morbidity variables used in estimating the factors associated with WTP were dichotomous. We opted to use dichotomous rather than continuous (i.e., percentage of days) morbidity variables for two primary reasons. First, for many of the morbidity variables, the average percentage of days the mothers/children experienced the morbidity event during the reference period was either very high or very low, resulting in highly skewed data with some extreme outliers. Second, translating the regression results into policy-relevant recommendations is more straightforward when the results can be applied to an easily identifiable group (e.g., mothers who have experienced nausea and vomiting during pregnancy) rather than as a marginal effect. However, we also recognize that information is lost by making the continuous morbidity variables discreet. To check the sensitivity of our estimated associations between the morbidity variables and WTP to the dichotomous classification of the morbidity variables, we reran the regressions in tables 2-4 of the main paper with all morbidity variables defined as the percentage of days in the reference period in which the mother/child experienced the morbidity event.

The results of this sensitivity analysis, shown in Tables A7-A9, show that the estimated associations were, with a few exceptions, qualitatively very similar. One exception is that in the pregnancy period in Ghana, maternal nausea or vomiting was no longer statistically significantly associated with WTP for a day’s supply of SQ-LNS using the continuous variable and was only marginally significant (p<.10) and quite small in magnitude in the long-term WTP equation. Another notable exception is that maternal poor appetite was statistically significantly related to WTP in Ghana in the postpartum period using the continuous variable; each additional percentage of days of reported maternal poor appetite during the reference period was associated with lower WTP for both a day’s supply and in the long-term by approximately $0.01 (p<.10), all else constant. In the child period in Malawi, while the dichotomous specifications of general infant illness and infant diarrhea were both associated with WTP, neither the percentage of days in the reference period that the infant was ill nor the percentage of days the infant experienced diarrhea were statistically significant.

We also tested the sensitivity of our results to the interval of time that defined the morbidity variables. Instead of taking a long-term look back at morbidity events as defined in Table 2 of the main paper, it could be that only the most immediate morbidity events were relevant in influencing WTP. To test the sensitivity of our results to the long- versus short-term morbidity reference periods, we redefined all reference periods as the 30 days period prior to WTP survey administration and again reran the regressions from tables 2-4 of the main paper. These results are shown in tables A10-12.

In the pregnancy period, the only notable difference in the results based on the shorter reference period was that whereas the association between maternal diarrhea and WTP was not statistically significant in Malawi based on the longer reference period, WTP for a day’s supply was approximately $0.08 lower (p<.05) if the mother reported experiencing diarrhea in the prior 30 days, all else constant. There were also several notable differences in the postpartum period. First, in Ghana, if mothers reported poor appetite in the 30 days prior, long-term WTP was approximately $0.13 lower (p<.05) all else constant, while the relationship was negative but not statistically significant based on the longer reference period. Also in Ghana, the marginally significant (p<.10) association between WTP for a day’s supply and both infant poor appetite and infant vomiting based on the longer reference period were not statistically significant when focusing on just the 30 days before the WTP survey was administered. However, WTP in the long-term was estimated to be $0.10 lower (p<.05) if the infant experienced vomiting in the 30 days before reporting WTP. In Malawi, maternal diarrhea in the 30 days before WTP survey administration was estimated to put downward pressure on WTP (p<.1), a relationship that was not significant based on the longer reference period. Finally, in the child period, the negative association between WTP for a day’s supply and infant diarrhea in Malawi based on the longer reference period was no longer statistically significant when focusing on infant diarrhea in the 30-day window before WTP survey administration.

Taken together, this set of sensitivity analyses suggests that our results were generally not sensitive to defining the morbidity variables as dichotomous vs continuous, though the estimated associations between the morbidity variables and WTP were a bit more sensitive to the duration of the reference period. Because some morbidity variables appeared more appropriately defined in the longer term, at least with respect to their association with WTP, while others appeared to be better captured by the shorter reference period, it is not obvious whether a shorter or longer reference period is most informative in this context.

Table A7. Factors Associated with WTP during the Pregnancy Period, Continuous Morbidity Variables

|  | Ghana | | Malawi |  |
| --- | --- | --- | --- | --- |
| Variable | Day’s Supply | Long-Term | Day’s Supply | |
| LNS Group (0/1) | -0.011 | 0.022 | 0.008 | |
|  | (0.045) | (0.034) | (0.039) | |
| Months from Enrollment | -0.017* | -0.030*** | 0.025* | |
|  | (0.010) | (0.006) | (0.013) | |
| Lean Season | -0.004 | -0.014 | 0.101*** | |
|  | (0.030) | (0.021) | (0.038) | |
| Mangochi (0/1) |  |  | 0.011 | |
|  |  |  | (0.026) | |
| Mother (0/1) | -0.147** | -0.081* |  | |
|  | (0.058) | (0.043) |  | |
| Respondent Age | -0.005** | -0.002 | 0.005 | |
|  | (0.002) | (0.002) | (0.004) | |
| Respondent Education | 0.003 | 0.006 | -0.003 | |
|  | (0.006) | (0.005) | (0.007) | |
| Asset Index | -0.030 | -0.008 | 0.021 | |
|  | (0.026) | (0.022) | (0.019) | |
| HFIAS Score | -0.016*** | -0.015*** | -0.009** | |
|  | (0.005) | (0.003) | (0.004) | |
| Primiparity (0/1) | -0.010 | -0.031 | -0.043 | |
|  | (0.050) | (0.040) | (0.035) | |
| Maternal Poor Appetite (%) | 0.002 | 0.001 | -0.000 | |
|  | (0.002) | (0.001) | (0.001) | |
| Maternal Nausea or Vomiting (%) | -0.001 | -0.001* | -0.001 | |
|  | (0.001) | (0.001) | (0.001) | |
| Maternal Diarrhea (%) | -0.000 | -0.001 | -0.001 | |
|  | (0.004) | (0.003) | (0.002) | |
| Constant | 0.805*** | 0.586*** | -0.002 | |
|  | (0.140) | (0.114) | (0.088) | |
| N | 971 | 966 | 889 | |
| Wald Chi^2^ | 32.006 | 83.560 | 26.702 | |

Significance codes: *** (p < .01), ** (p < .05), * (p < .1).

Notes: Dependent variables are stated WTP in 2011 US dollars. Controls for randomized starting bid are included in all models (unreported). Standard errors, in parentheses, obtained via 50 bootstrap replications.

Table A8. Factors Associated with WTP during the Postpartum Period, Continuous Morbidity Variables

|  | Ghana | | Malawi |  |
| --- | --- | --- | --- | --- |
| Variable | Day’s Supply | Long-Term | Day’s Supply | |
| LNS Group (0/1) | 0.018 | 0.011 | 0.032 | |
|  | (0.039) | (0.030) | (0.025) | |
| Months from Birth | 0.039 | 0.012 | -0.054** | |
|  | (0.024) | (0.022) | (0.027) | |
| Lean Season | 0.034 | 0.040 | -0.019 | |
|  | (0.035) | (0.029) | (0.022) | |
| Mangochi (0/1) |  |  | -0.001 | |
|  |  |  | (0.026) | |
| Mother (0/1) | -0.140*** | -0.092*** |  | |
|  | (0.041) | (0.035) |  | |
| Respondent Age | -0.002 | -0.001 | 0.003 | |
|  | (0.002) | (0.001) | (0.002) | |
| Respondent Education | 0.001 | 0.003 | 0.002 | |
|  | (0.005) | (0.004) | (0.004) | |
| Asset Index | -0.003 | -0.002 | 0.027* | |
|  | (0.018) | (0.014) | (0.014) | |
| HFIAS Score | -0.017*** | -0.019*** | -0.003 | |
|  | (0.006) | (0.004) | (0.003) | |
| Primiparity (0/1) | -0.019 | -0.010 | 0.019 | |
|  | (0.035) | (0.029) | (0.039) | |
| Maternal Poor Appetite (%) | -0.006* | -0.006* | 0.000 | |
|  | (0.003) | (0.003) | (0.002) | |
| Maternal Diarrhea (%) | 0.016 | 0.022 | -0.001 | |
|  | (0.016) | (0.016) | (0.003) | |
| Infant Ill (%) | -0.003 | -0.002 | -0.000 | |
|  | (0.003) | (0.003) | (0.001) | |
| Infant Poor Appetite (%) | -0.002 | -0.002 | 0.002 | |
|  | (0.006) | (0.005) | (0.001) | |
| Infant Diarrhea (%) | 0.001 | 0.002 | 0.004 | |
|  | (0.004) | (0.003) | (0.004) | |
| Infant Vomiting (%) | 0.001 | -0.001 | -0.001 | |
|  | (0.002) | (0.002) | (0.001) | |
| Infant Male (0/1) | 0.100*** | 0.050* | 0.027 | |
|  | (0.034) | (0.028) | (0.023) | |
| BMIZ at Birth | 0.045*** | 0.037*** | -0.009 | |
|  | (0.017) | (0.013) | (0.012) | |
| LAZ at Birth | -0.018 | -0.021 | -0.005 | |
|  | (0.019) | (0.015) | (0.011) | |
| Constant | 0.441*** | 0.383*** | 0.237** | |
|  | (0.137) | (0.118) | (0.106) | |
| N | 526 | 526 | 338 | |
| Pseudo R^2^ | 0.093 | 0.138 | 1.329 | |

Significance codes: *** (p < .01), ** (p < .05), * (p < .1).

Notes: Dependent variables are stated WTP in 2011 US dollars. Controls for randomized starting bid are included in all models (unreported). Robust standard errors in parentheses.

Table A9. Factors Associated with WTP during the Child Period, Continuous Morbidity Variables

|  | Ghana | | Malawi |  |
| --- | --- | --- | --- | --- |
| Variable | Day’s Supply | Long-Term | Day’s Supply | |
| LNS Group (0/1) | 0.003 | -0.007 | -0.016 | |
|  | (0.034) | (0.019) | (0.020) | |
| Months from Birth | -0.008 | -0.006 | -0.009*** | |
|  | (0.005) | (0.004) | (0.002) | |
| Lean Season | 0.019 | -0.001 | -0.033 | |
|  | (0.031) | (0.015) | (0.021) | |
| Mangochi (0/1) |  |  | 0.024 | |
|  |  |  | (0.027) | |
| Mother (0/1) | -0.123*** | -0.085*** |  | |
|  | (0.030) | (0.024) |  | |
| Respondent Age | 0.000 | -0.000 | 0.002 | |
|  | (0.002) | (0.001) | (0.002) | |
| Respondent Education | 0.004 | 0.003 | -0.004 | |
|  | (0.003) | (0.003) | (0.004) | |
| Asset Index | -0.007 | 0.003 | 0.041*** | |
|  | (0.013) | (0.009) | (0.016) | |
| HFIAS Score | -0.015*** | -0.011*** | -0.006*** | |
|  | (0.004) | (0.002) | (0.002) | |
| Primiparity (0/1) | -0.029 | -0.028 | 0.017 | |
|  | (0.034) | (0.020) | (0.021) | |
| Infant Ill (%) | -0.001 | -0.000 | 0.002 | |
|  | (0.003) | (0.001) | (0.001) | |
| Infant Poor Appetite (%) | -0.001 | 0.000 | 0.000 | |
|  | (0.001) | (0.001) | (0.001) | |
| Infant Diarrhea (%) | -0.000 | -0.000 | -0.002 | |
|  | (0.002) | (0.001) | (0.001) | |
| Infant Vomiting (%) | 0.002 | -0.001 | 0.001 | |
|  | (0.005) | (0.003) | (0.002) | |
| Infant Male (0/1) | 0.093*** | 0.049*** | -0.011 | |
|  | (0.032) | (0.017) | (0.020) | |
| LAZ | -0.006 | 0.007 | -0.003 | |
|  | (0.016) | (0.014) | (0.007) | |
| WLZ | 0.001 | 0.009 | 0.014 | |
|  | (0.013) | (0.007) | (0.013) | |
| Constant | 0.445*** | 0.412*** | 0.189*** | |
|  | (0.105) | (0.093) | (0.060) | |
| N | 732 | 731 | 710 | |
| Wald Chi^2^ | 98.187 | 158.244 | 96.794 | |

Significance codes: *** (p < .01), ** (p < .05), * (p < .1).

Notes: Dependent variables are stated WTP in 2011 US dollars. Controls randomized starting bid are included in all models (unreported). Standard errors, in parentheses, obtained via 50 bootstrap replications.

Table A10. Factors Associated with WTP during the Pregnancy Period, 30-day Reference Period

|  | Ghana | | Malawi |  |
| --- | --- | --- | --- | --- |
| Variable | Day’s Supply | Long-Term | Day’s Supply | |
| LNS Group (0/1) | 0.000 | 0.027 | 0.004 | |
|  | (0.044) | (0.032) | (0.032) | |
| Months from Enrollment | 0.001 | -0.022** | 0.027* | |
|  | (0.017) | (0.009) | (0.016) | |
| Lean Season | -0.011 | -0.022 | 0.098** | |
|  | (0.033) | (0.023) | (0.040) | |
| Mangochi (0/1) |  |  | 0.010 | |
|  |  |  | (0.038) | |
| Mother (0/1) | -0.140*** | -0.081* |  | |
|  | (0.044) | (0.042) |  | |
| Respondent Age | -0.005** | -0.002 | 0.005* | |
|  | (0.003) | (0.002) | (0.003) | |
| Respondent Education | 0.003 | 0.006 | -0.005 | |
|  | (0.006) | (0.004) | (0.007) | |
| Asset Index | -0.031 | -0.010 | 0.022 | |
|  | (0.023) | (0.019) | (0.020) | |
| HFIAS Score | -0.015*** | -0.014*** | -0.009** | |
|  | (0.005) | (0.004) | (0.004) | |
| Primiparity (0/1) | -0.008 | -0.030 | -0.049 | |
|  | (0.042) | (0.031) | (0.036) | |
| Maternal Poor Appetite (0/1) | 0.016 | -0.005 | -0.019 | |
|  | (0.054) | (0.035) | (0.029) | |
| Maternal Nausea or Vomiting (0/1) | -0.078* | -0.081** | -0.005 | |
|  | (0.046) | (0.032) | (0.026) | |
| Maternal Diarrhea (0/1) | 0.116 | 0.015 | -0.078** | |
|  | (0.072) | (0.046) | (0.039) | |
| Constant | 0.909*** | 0.614*** | 0.021 | |
|  | (0.184) | (0.130) | (0.071) | |
| N | 966 | 961 | 882 | |
| Wald Chi^2^ | 63.469 | 72.390 | 49.680 | |

Significance codes: *** (p < .01), ** (p < .05), * (p < .1).

Notes: Dependent variables are stated WTP in 2011 US dollars. Controls for number of days in reference period for calculation of morbidity variables and indicators for randomized starting bid are included in all models (unreported). Standard errors, in parentheses, obtained via 50 bootstrap replications.

Table A11. Factors Associated with WTP during the Postpartum Period, 30-day Reference Period

|  | Ghana | | Malawi |  |
| --- | --- | --- | --- | --- |
| Variable | Day’s Supply | Long-Term | Day’s Supply | |
| LNS Group (0/1) | 0.009 | 0.013 | 0.039 | |
|  | (0.039) | (0.030) | (0.025) | |
| Months from Birth | 0.030 | 0.004 | -0.056** | |
|  | (0.023) | (0.020) | (0.026) | |
| Lean Season | 0.035 | 0.045 | -0.014 | |
|  | (0.035) | (0.029) | (0.022) | |
| Mangochi (0/1) |  |  | 0.006 | |
|  |  |  | (0.028) | |
| Mother (0/1) | -0.138*** | -0.089** |  | |
|  | (0.041) | (0.035) |  | |
| Respondent Age | -0.002 | -0.001 | 0.003 | |
|  | (0.002) | (0.001) | (0.002) | |
| Respondent Education | -0.001 | 0.002 | 0.001 | |
|  | (0.005) | (0.004) | (0.004) | |
| Asset Index | 0.001 | 0.004 | 0.027* | |
|  | (0.017) | (0.014) | (0.014) | |
| HFIAS Score | -0.017*** | -0.019*** | -0.003 | |
|  | (0.006) | (0.004) | (0.003) | |
| Primiparity (0/1) | -0.029 | -0.024 | 0.010 | |
|  | (0.036) | (0.030) | (0.039) | |
| Maternal Poor Appetite (0/1) | -0.099 | -0.126** | -0.052 | |
|  | (0.062) | (0.051) | (0.061) | |
| Maternal Diarrhea (0/1) | -0.015 | 0.065 | -0.093* | |
|  | (0.082) | (0.075) | (0.053) | |
| Infant Ill (0/1) | 0.029 | -0.001 | 0.013 | |
|  | (0.056) | (0.049) | (0.028) | |
| Infant Poor Appetite (0/1) | -0.074 | -0.022 | 0.033 | |
|  | (0.071) | (0.058) | (0.046) | |
| Infant Diarrhea (0/1) | -0.058 | -0.013 | 0.018 | |
|  | (0.053) | (0.047) | (0.035) | |
| Infant Vomiting (0/1) | -0.073 | -0.097** | -0.099 | |
|  | (0.057) | (0.040) | (0.063) | |
| Infant Male (0/1) | 0.093*** | 0.041 | 0.023 | |
|  | (0.035) | (0.029) | (0.023) | |
| BMIZ at Birth | 0.046*** | 0.036*** | -0.002 | |
|  | (0.017) | (0.013) | (0.012) | |
| LAZ at Birth | -0.018 | -0.023 | -0.008 | |
|  | (0.020) | (0.016) | (0.012) | |
| Constant | 0.337 | 0.481** | 0.174 | |
|  | (0.257) | (0.221) | (0.115) | |
| N | 517 | 517 | 331 | |
| Pseudo R^2^ | 0.093 | 0.142 | 1.044 | |

Significance codes: *** (p < .01), ** (p < .05), * (p < .1).

Notes: Dependent variables are stated WTP in 2011 US dollars. Controls for number of days in reference period for calculation of morbidity variables and indicators for randomized starting bid are included in all models (unreported). Robust standard errors in parentheses.

Table A12. Factors Associated with WTP during the Child Period, 30-day Reference Period

|  | Ghana | | Malawi |  |
| --- | --- | --- | --- | --- |
| Variable | Day’s Supply | Long-Term | Day’s Supply | |
| LNS Group (0/1) | 0.003 | -0.007 | -0.014 | |
|  | (0.034) | (0.018) | (0.017) | |
| Months from Birth | -0.007 | -0.006 | -0.009*** | |
|  | (0.005) | (0.004) | (0.002) | |
| Lean Season | 0.018 | -0.002 | -0.034 | |
|  | (0.032) | (0.015) | (0.021) | |
| Mangochi (0/1) |  |  | 0.022 | |
|  |  |  | (0.019) | |
| Mother (0/1) | -0.121*** | -0.083*** |  | |
|  | (0.029) | (0.024) |  | |
| Respondent Age | 0.000 | -0.000 | 0.002 | |
|  | (0.002) | (0.001) | (0.002) | |
| Respondent Education | 0.004 | 0.003 | -0.004 | |
|  | (0.003) | (0.003) | (0.004) | |
| Asset Index | -0.009 | 0.003 | 0.040** | |
|  | (0.013) | (0.009) | (0.016) | |
| HFIAS Score | -0.015*** | -0.011*** | -0.006*** | |
|  | (0.004) | (0.002) | (0.002) | |
| Primiparity (0/1) | -0.029 | -0.028 | 0.017 | |
|  | (0.034) | (0.019) | (0.024) | |
| Infant Ill (0/1) | 0.033 | 0.036 | 0.055*** | |
|  | (0.047) | (0.024) | (0.019) | |
| Infant Poor Appetite (0/1) | -0.041 | -0.032 | -0.023 | |
|  | (0.035) | (0.023) | (0.022) | |
| Infant Diarrhea (0/1) | -0.006 | 0.000 | -0.021 | |
|  | (0.035) | (0.020) | (0.021) | |
| Infant Vomiting (0/1) | -0.021 | -0.030 | -0.027 | |
|  | (0.052) | (0.029) | (0.052) | |
| Infant Male (0/1) | 0.094*** | 0.051*** | -0.007 | |
|  | (0.032) | (0.017) | (0.018) | |
| LAZ | -0.007 | 0.006 | -0.001 | |
|  | (0.017) | (0.013) | (0.008) | |
| WLZ | 0.003 | 0.009 | 0.013 | |
|  | (0.012) | (0.007) | (0.010) | |
| Constant | 0.314** | 0.370*** | 0.189*** | |
|  | (0.127) | (0.106) | (0.052) | |
| N | 733 | 732 | 706 | |
| Wald Chi^2^ | 121.041 | 175.555 | 104.267 | |

Significance codes: *** (p < .01), ** (p < .05), * (p < .1).

Notes: Dependent variables are stated WTP in 2011 US dollars. Controls for number of days in reference period for calculation of morbidity variables and indicators for randomized starting bid are included in all models (unreported). Standard errors, in parentheses, obtained via 50 bootstrap replications.

**References**

Adu-Afarwuah, Seth, Anna Lartey, Harriet Okronipa, Per Ashorn, Janet M Peerson, Mary Arimond, Ulla Ashorn, Mamane Zeilani, Stephen Vosti, and Kathryn G Dewey. 2016. "Small-Quantity, Lipid-Based Nutrient Supplements Provided to Women During Pregnancy and 6 mo Postpartum and to their Infants fom 6 mo of Age Increase the Mean Attained Length of 18-mo-old Children in Semi-Urban Ghana: A Randomized Controlled Trial." *The American Journal of Clinical Nutrition,* 103(3): 797-808.

Adu-Afarwuah, Seth, Anna Lartey, Harriet Okronipa, Per Ashorn, Mamane Zeilani, Janet M Peerson, Mary Arimond, Stephen Vosti, and Kathryn G Dewey. 2015. "Lipid-Based Nutrient Supplement Increases the Birth Size of Infants of Primiparous Women in Ghana." *The American Journal of Clinical Nutrition,* 101(4): 835-846.

1. In Malawi, respondents were asked about their willingness to pay for a week’s supply. For purposes of cross-site comparison, WTP for a week’s supply has been converted to a daily rate for all analyses. [↑](#footnote-ref-1)
2. In Ghana, the dichotomous choice question was, “If you went to the new kiosk today and the owner quoted you a price of GH¢ X for 1 sachet of nkatepa [local name for SQ-LNS], which is 20 grams, would you purchase it, bearing in mind your income and daily expenses?” In Malawi, the question was “If Chiponde chatsopano [local name for SQ-LNS], was available from the new kiosk for K X for a week’s supply, would you purchase it?” [↑](#footnote-ref-2)
3. The randomized starting bids for a day’s supply in Ghana were GH¢ 0.20, GH¢ 0.50, or GH¢ 1.00 (approximately US $0.13, $0.33, or $0.66). The randomized starting bids for a week’s supply (seven sachets) in Malawi were K100, K200, or K300 (approximately US $0.30, $0.60, or $0.90). [↑](#footnote-ref-3)
4. For WTP for LNS-P&L in Ghana, these follow-up questions began with the following: “You have told me that you would be willing to pay [maximum WTP] today for one sachet of nkatepa. Would you be willing to pay [maximum WTP] per day for 1 sachet of nkatepa throughout your pregnancy?” If the answer was ‘no’, then the following was asked: “What price do you think you could pay every day for 1 sachet of nkatepa throughout your pregnancy?” For WTP for LNS-Child, the follow-up questions began with: “You have told me that you would be willing to pay [maximum WTP] today for 2 sachets of nkatepa junior, each containing 10grams. Would you be willing to pay [maximum WTP] for 2 sachets of nkatepa junior every day when the iLiNS child is between the ages of 6 months and approximately 18 months?” [↑](#footnote-ref-4)
5. As a comparator to WTP for SQ-LNS, each time WTP for LNS-P&L/LNS-Child was collected, we also elicited WTP for a day’s supply of a locally-available product commonly used to enhance the nutrient content of traditional diets. In Ghana the local product was soybean flour, which is typically sold by nurses to women attending prenatal and well-baby clinics in the iLiNS study area. In Malawi, the local product for mothers was bonya, a small dried fish. The local product for children was Likuni Phala, a fortified corn-soy blend. [↑](#footnote-ref-5)
6. In cases where the mother was also the head of household, this variable was coded as Mother = 1. [↑](#footnote-ref-6)
7. From a household perspective, these benefits might include things that are important from a nutritional perspective (e.g., birth outcomes, linear growth, and development) but may also include other benefits attributable to sensory appeal, feelings of protecting the well-being of the infant, etc. [↑](#footnote-ref-7)
